# Transcriptome analysis reveals differential transcription in tomato (*Solanum lycopersicum*) following inoculation with *Ralstonia solanacearum*

**DOI:** 10.1038/s41598-022-26693-y

**Published:** 2022-12-22

**Authors:** Na Chen, Qin Shao, Qineng Lu, Xiaopeng Li, Yang Gao

**Affiliations:** College of Life Science and Resources and Environment, Yichun University, Yichun, 336000 China

**Keywords:** Biological techniques, Molecular biology, Plant sciences

## Abstract

Tomato (*Solanum lycopersicum* L.) is a major Solanaceae crop worldwide and is vulnerable to bacterial wilt (BW) caused by *Ralstonia solanacearum* during the production process. BW has become a growing concern that could enormously deplete the tomato yield from 50 to 100% and decrease the quality. Research on the molecular mechanism of tomato regulating BW resistance is still limited. In this study, two tomato inbred lines (Hm 2–2, resistant to BW; and BY 1–2, susceptible to BW) were used to explore the molecular mechanism of tomato in response to *R. solanacearum* infection by RNA-sequencing (RNA-seq) technology. We identified 1923 differentially expressed genes (DEGs) between Hm 2–2 and BY 1–2 after *R. solanacearum* inoculation. Among these DEGs, 828 were up-regulated while 1095 were down-regulated in R-3dpi (Hm 2–2 at 3 days post-inoculation with *R. solanacearum*) vs. R-mock (mock-inoculated Hm 2–2); 1087 and 2187 were up- and down-regulated, respectively, in S-3dpi (BY 1–2 at 3 days post-inoculation with *R. solanacearum*) vs. S-mock (mock-inoculated BY 1–2). Moreover, Gene Ontology (GO) enrichment analysis revealed that the largest amount of DEGs were annotated with the Biological Process terms, followed by Cellular Component and Molecular Function terms. A total of 114, 124, 85, and 89 regulated (or altered) pathways were identified in R-3dpi vs. R-mock, S-3dpi vs. S-mock, R-mock vs. S-mock, and R-3dpi vs. S-3dpi comparisons, respectively, by Kyoto Encyclopaedia of Genes and Genomes (KEGG) pathway analysis. These clarified the molecular function and resistance pathways of DEGs. Furthermore, quantitative RT-PCR (qRT-PCR) analysis confirmed the expression patterns of eight randomly selected DEGs, which suggested that the RNA-seq results were reliable. Subsequently, in order to further verify the reliability of the transcriptome data and the accuracy of qRT-PCR results, *WRKY75*, one of the eight DEGs was silenced by virus-induced gene silencing (VIGS) and the defense response of plants to *R. solanacearum* infection was analyzed. In conclusion, the findings of this study provide profound insight into the potential mechanism of tomato in response to *R. solanacearum* infection, which lays an important foundation for future studies on BW.

## Introduction

Tomato (*Solanum lycopersicum* L.) is one of the majorly consumed solanaceous vegetable crops, with a global annual yield of approximately 160 million tons^1,2^. It is cultivated worldwide for fresh vegetable consumption as well as for industrial processing^3,4^. Tomatoes are nutritionally significant that can provide vitamins, fibers, and minerals. They are essential sources of β-carotene and lycopene possessing antioxidant and have anti-cancer properties^5^. A large part of the tomato crop has been grown continuously for many years in the world. Continuous tomato cultivation in China has led to an outbreak of the devastating soil-borne disease "bacterial wilt" (BW), caused by virulent *Ralstonia solanacearum*. BW can enormously cause the production loss by 50–100% every year, and it has become one of the main diseases that seriously threaten the yield and quality of tomato^6–9^. BW is one of the most serious plant diseases in the world^10,11^. It is a typical vascular disease that harms the roots, stems, and leaves. BW has a rapid onset, withered leaves on the diseased side, wilted leaves, and striped diseased spots at the base of the stem. In severe cases, the roots would turn black and rot, and the pith would be hollow or honeycomb. When the diseased plant is cut open, the fibrous tube tissue inside will turn brown. When the transverse cut of the diseased plant base is pressed hard, the yellow-white bacterial mucus will flow out of the fracture, which is known as "bacteria pus"^12,13^. Bacterial wilt, known as plant cancer, seriously affects the yield and quality of crops. It has strong variability and soil-borne characteristics. The current traditional control methods, such as breeding resistant varieties, crop rotation and chemical control have some limitations^14–16^. Therefore, it is necessary to have a comprehensive and detailed understanding of plant–pathogen interactions during the progression of BW^17^.

The current research on tomato resistance to BW mainly focuses on the genetic mechanism of resistance^18–21^; identification^20,22,23^ and screening^13,24–29^ of molecular markers related to the disease resistance genes and other aspects. There are only a few studies available on the gene regulation of BW resistance^30–37^, and the molecular mechanism of tomato resistance-related genes regulating BW remains unclear. Thus, it necessitates performing the research on the response of tomato plant to BW. In recent years, with the application of transcriptome sequencing technology, several genes and miRNA functions have been identified. Transcriptome sequencing has been widely used in basic research, molecular breeding, pathogen-host interaction mechanism, comparison of resistance genes between susceptible and disease-resistant varieties, and biocontrol factors inducing plant disease-resistance mechanisms^38–40^. Transcriptomic technique has been used in many studies to identify the molecular mechanism of *R. solanacearum* resistance in a plethora of plant species, including *Solanum dulcamara*^41^, *Arachis hypogaea*^42^, *Solanum commersonii*^43^, *Solanum melongena*^44^, *Capsicum annuum*^45^, *Solanum tuberosum*^46^, and *Nicotiana tabacum*^47^. In tomato, the in-depth transcriptome data is available for its interaction with *R. solanacearum*^15,17,48–50^. French et al.^48^ analyzed the genome-wide transcriptional response of roots of resistant and susceptible tomato plants at multiple time points after inoculation with *R. solanacearum* and identified the molecular basis of this resistance. Furthermore, they determined the role of auxin signaling and transport pathways in resistance against *R. solanacearum* by functional analysis of an auxin transport tomato mutant. Jiang et al.^49^ investigated the root transcriptome profiles between silicon (Si)-treated (+ Si) and untreated (− Si) tomato plants at different days post-inoculation with *R. solanacearum* by using RNA-seq technology. They also determined the content of hormones including salicylic acid (SA), jasmonic acid (JA), and ethylene (ET). Finally, they suggested that Si enhanced BW resistance of tomato via several signaling pathol.ways. The molecular mechanism of tomato resistance-related genes regulating bacterial wilt resistance is still unclear. Therefore, it is necessary to screen out the genes related to tomato BW resistance through transcriptome analysis.

In this study, we used RNA-seq technology to analyze the transcriptome of two tomato inbred lines resistant to BW Hm 2–2 (R) and susceptible to BW BY 1–2 (S) before and after inoculation with *R. solanacearum*. The two tomato inbred lines are special and the inductive properties are stable. In addition, studies comfirmed that *Ralstonia solanacearum* can multiply in plant stems. In this study, tomato stems were collected and studied, while tomato roots were studied in most previous studies. This study aimed to determine the molecular mechanism involved in the tomato response towards *R. solanacearum*, and provide a theoretical foundation for future research in BW.

## Results

### Phenotypic characterization after inoculation with *R. solanacearum*

**Figure 1:**
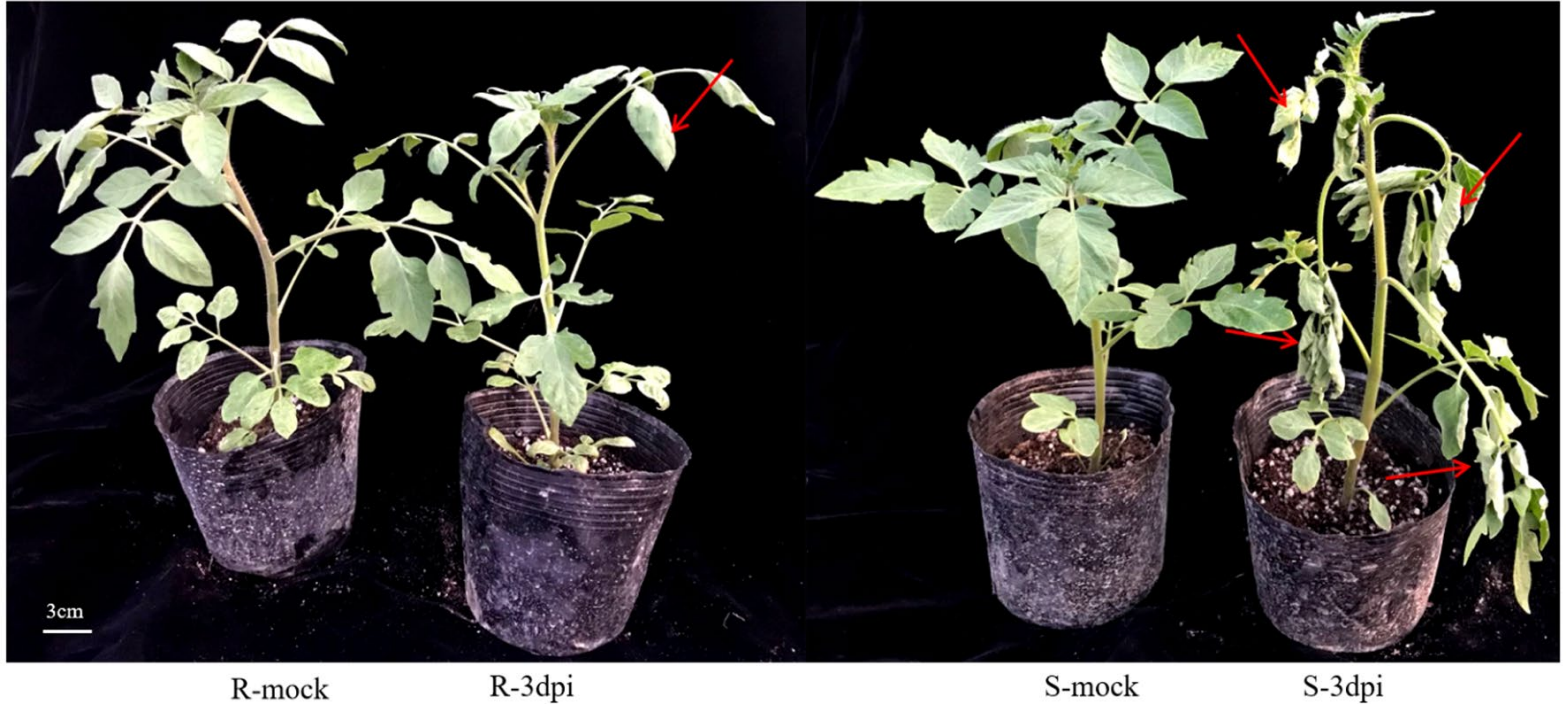
Phenotypic symptoms of resistant (Hm 2–2) and susceptible (BY 1–2) tomato seedlings 3 days after *Ralstonia solanacearum* inoculation. R-mock represents mock-inoculated Hm 2–2 plants; R-3dpi represents 3 days post-pathogen-inoculated Hm 2–2 plants; S-mock represents mock-inoculated BY 1–2 plants; S-3dpi represents 3 days post-pathogen-inoculated BY 1–2 plants. The red arrows represent wilting symptoms of tomato leaves.

At 3 dpi (days post-inoculation), plants exhibited different phenotypic symptoms. As shown in Fig. 1, leaves of the resistant plants (Hm 2–2) and susceptible (BY 1–2) plants inoculated with sterile water showed no obvious symptoms. However, one or two leaves of the resistant plants (Hm 2–2) inoculated with pathogen showed wilting symptoms, but almost all the leaves of the susceptible (BY 1–2) plants inoculated with pathogen showed wilting symptoms. These results indicate that tomato Hm 2–2 and BY 1–2 plants respond differently to *R. solanacearum* infection.

### Analysis of RNA-seq data

To obtain a global overview of the transcriptome relevant to BW stress conditions in the resistant and susceptible tomato plants, the cDNA libraries from stem samples of Hm 2–2 and BY 1–2 plants with mock-inoculation and pathogen-inoculation were sequenced on the Illumina HiSeq2500 platform, separately. After removal of the adaptors and filtration of low-quality reads, an average of 3.3 × 10^7^ clean reads were obtained for each sample, and clean reads from all twelve libraries were mapped to the tomato genome ITAG3.2. The coverage of overall mapped reads to all the samples ranged between 96–97% and the percentage of uniquely mapped reads ranged between 76 and 85%. Moreover, the percentage of bases with Q20 (high sequencing quality) was close to 98%. Detailed sequencing and mapping statistics are given in Supplementary Table S1. As shown in Supplementary Fig. S1, gene coverage ranged from 80 to 100%, accounting for approximately 75% of the total genes (Supplementary Fig. S1a). An experimental repeatability test was performed, the results of which indicated that the results between replicates were comparable (Supplementary Fig. S1b). These results showed that the transcriptome sequencing quality was appropriate for further analyses.

### Identification of DEGs

**Figure 2:**
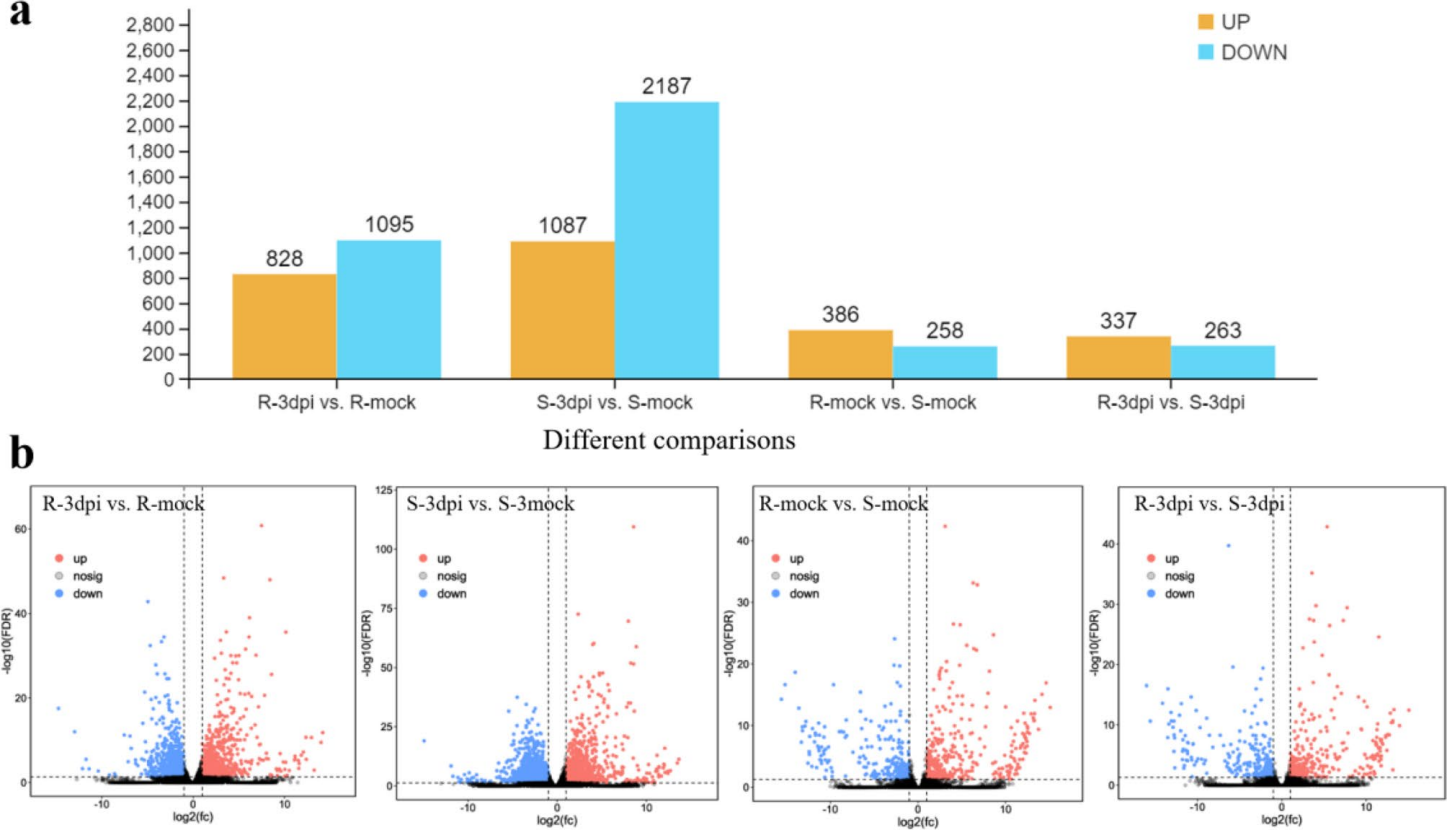
Differential expression analysis between treatments. (**a**) Comparison of the number of up- and down-regulated genes. Yellow and blue points represent up- and down-regulated genes, respectively. (**b**) Volcano plots between treatments and control. Red and blue points represent up- and down-regulated genes, respectively. Gray points represent no differential expression between genes. R-mock represents mock-inoculated Hm 2–2 plants; R-3dpi represents 3 days post-pathogen-inoculated Hm 2–2 plants; S-mock represents mock-inoculated BY 1–2 plants; S-3dpi represents 3 days post-pathogen-inoculated BY 1–2 plants.

Differential expression analysis was performed by the DESeq2 package between two groups (treatments and control). The genes with FDR < 0.05 and |log2FC|≥ 1 were considered DEGs. The number of DEGs was different in each of the four comparisons (Fig. 2a). The most DEGs were found in S-3dpi vs. S-mock (1087 and 2187 significantly up- and down-regulated genes, respectively), followed by R-3dpi vs. R-mock (828 and 1095 significantly up- and down-regulated genes, respectively) and R-mock vs. S-mock (386 and 258 significantly up- and down-regulated genes, respectively), and the least DEGs were seen in R-3dpi vs. S-3dpi (337 and 263 significantly up- and down-regulated genes, respectively). In R-3dpi vs. R-mock and S-3dpi vs. S-mock, the total number of down-regulated genes was greater than that of up-regulated genes. While in R-mock vs. S-mock and R-3dpi vs. S-3dpi, the total number of up-regulated genes was greater than that of down-regulated genes. The volcano plots showed that the number of up- and down-regulated genes had a clear distribution pattern in all four comparisons (Fig. 2b). Moreover, the distribution pattern of down-regulated genes in S-3dpi vs. S-mock was much higher than that in R-mock vs. S-mock and R-3dpi vs. S-3dpi. Although the numbers of up- and down-regulated genes in R-mock vs. S-mock and R-3dpi vs. S-3dpi were lower compared with those of the other two comparisons, the distribution patterns were very similar. These results clearly showed the global gene expression patterns between different comparisons.

**Figure 3:**
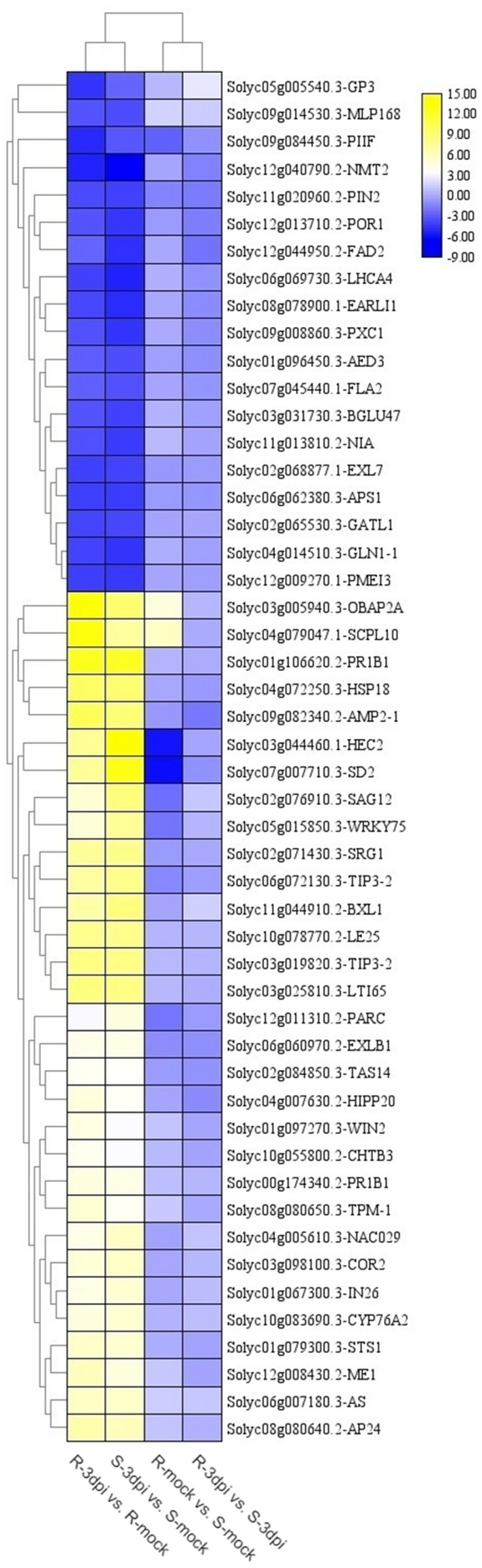
Heat map showing a hierarchical cluster analysis of the top 50 highly expressed genes in four comparisons. The gradient scale represents expression levels with yellow indicating the highest expression and blue indicating the lowest expression. R-mock represents mock-inoculated Hm 2–2 plants; R-3dpi represents 3 days post-pathogen-inoculated Hm 2–2 plants; S-mock represents mock-inoculated BY 1–2 plants; S-3dpi represents 3 days post-pathogen-inoculated BY 1–2 plants.

The heat map analysis based on hierarchical clustering analysis of DEGs in four comparisons showed that DEGs in different comparisons showed different expression trends. Among the top 50 highly correlated genes across samples, 31/19 clustered together to reveal higher up-/down-regulating trends in R-3dpi vs. R-mock and S-3dpi vs. S-mock comparisons. Most of the top 50 highly correlated genes exhibited little difference between R-mock vs. S-mock and R-3dpi vs. S-3dpi (Fig. 3). The expression of the top 50 DEGs in different comparisons is listed in Supplementary Table S3.

### SNP and InDel annotations

**Figure 4:**
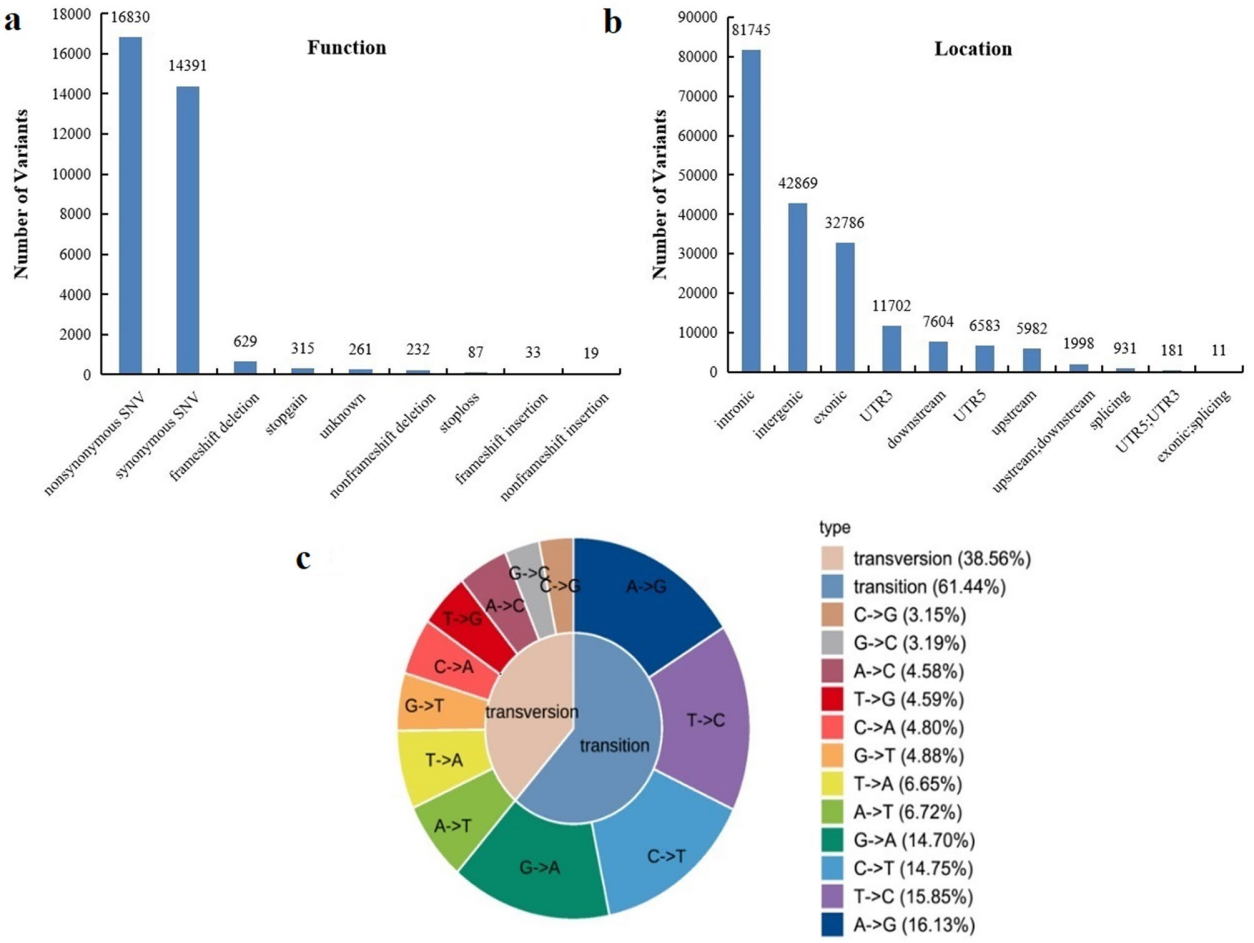
SNP/InDel annotation in terms of function (**a**), location (**b**), and type (**c**).

Variation detection based on SNPs and InDels of transcriptome sequencing was performed by the software GATKv3.4-46 (Fig. 4). Two types of nonsynonymous single nucleotide variation (SNV) and synonymous SNV represented dominant functional variations among the nine types of functional variations (Fig. 4a). The remaining types were frameshift deletion, stop gain, unknown, non-frameshift deletion stoploss, frameshift insertion, and non-frameshift insertion. Furthermore, these SNPs were distributed in different locations, with dominant distribution in intronic regions, followed by intergenic and exonic regions (Fig. 4b). We also identified all the possible mutations in this study. The two dominant types were transition and transversion (Fig. 4c), where transition accounted for 61.44%, while transversion accounted for 38.56%. These results suggest systematic, comprehensive, transcriptional regulation in tomato that orchestrates the response to BW.

### GO enrichment analysis of DEGs

**Figure 5:**
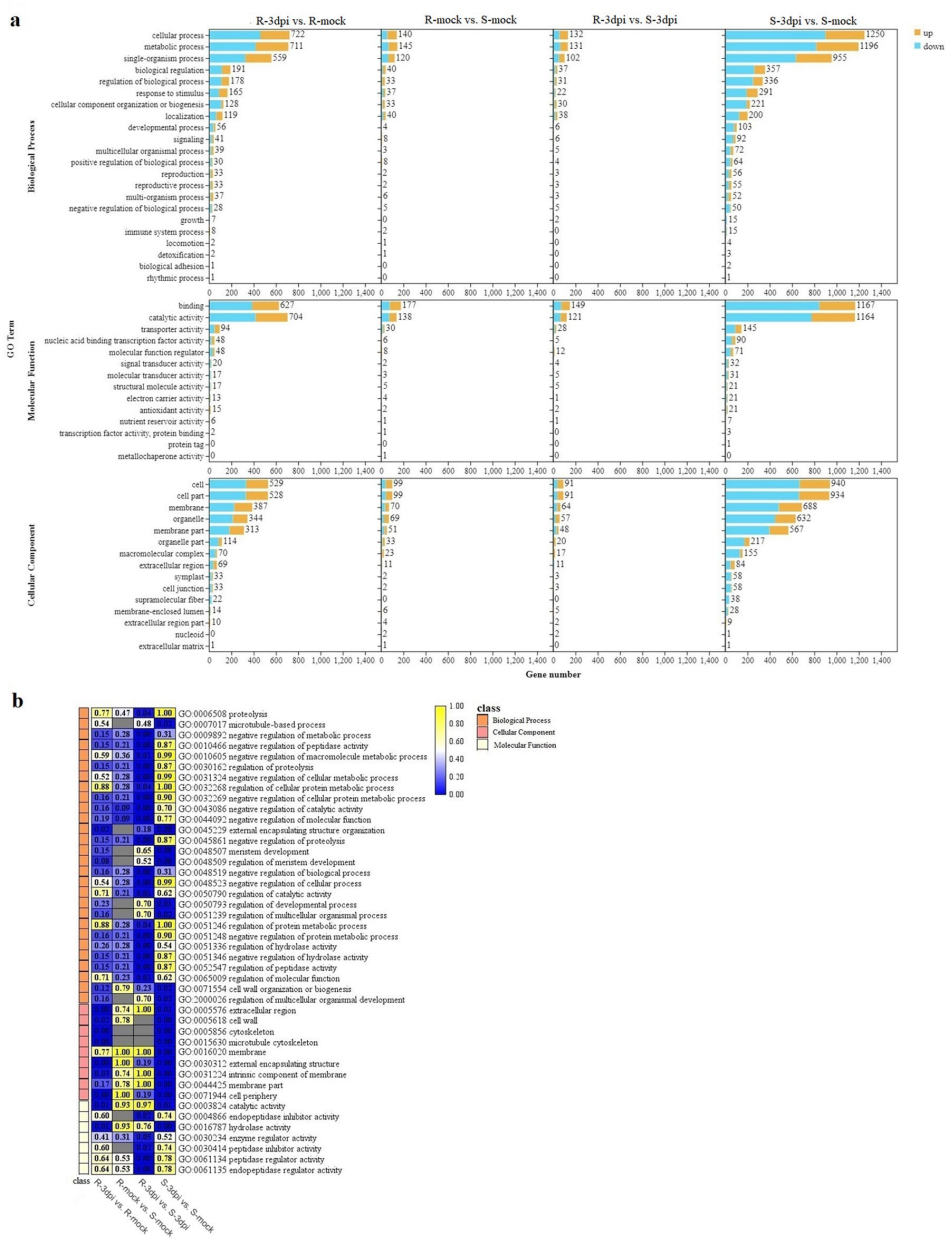
GO enrichment analyses of DEGs in four comparisons (R-3dpi vs. R-mock, R-mock vs. S-mock, R-3dpi vs. S-3dpi, and S-3dpi vs. S-mock). (**a**) Summary of the distribution and number of DEGs in three ontology classes, including biological process, molecular function, and cellular component. (**b**) Q-value heat map of the GO enrichment of the three main ontology classes. The color scale indicates the Q-value. Darker coloration indicates more significant enrichment. R-mock represents mock-inoculated Hm 2–2 plants; R-3dpi represents 3 days post-pathogen-inoculated Hm 2–2 plants; S-mock represents mock-inoculated BY 1–2 plants; S-3dpi represents 3 days post-pathogen-inoculated BY 1–2 plants.

GO enrichment analyses were performed for all significant DEGs (Fig. 5). Different comparisons showed similar distribution patterns in terms of the number and type of enriched GO terms, which could be divided into three main functional groups, including 22 biological processes, 14 molecular functions, and 15 cellular components (Fig. 5a). The largest amount of DEGs were annotated with the biological process terms, with the cellular process, metabolic process, and single-organism process accounting for the largest number of DEGs. In molecular function terms, the majority of DEGs were associated with binding and catalytic activities. Other significantly abundant cellular component terms were cell, cell part, membrane, organelle, and membrane parts (Supplementary Table S4). However, the enrichment level (Q-value) for each functional comparison varied (Fig. 5b). Notably, other functional comparisons that were significantly enriched were involved in different cellular component pathways, such as extracellular region, cytoskeleton, microtubule cytoskeleton, external encapsulating structure, and cell periphery processes (Fig. 5b).

### KEGG pathway analysis of DEGs

**Figure 6:**
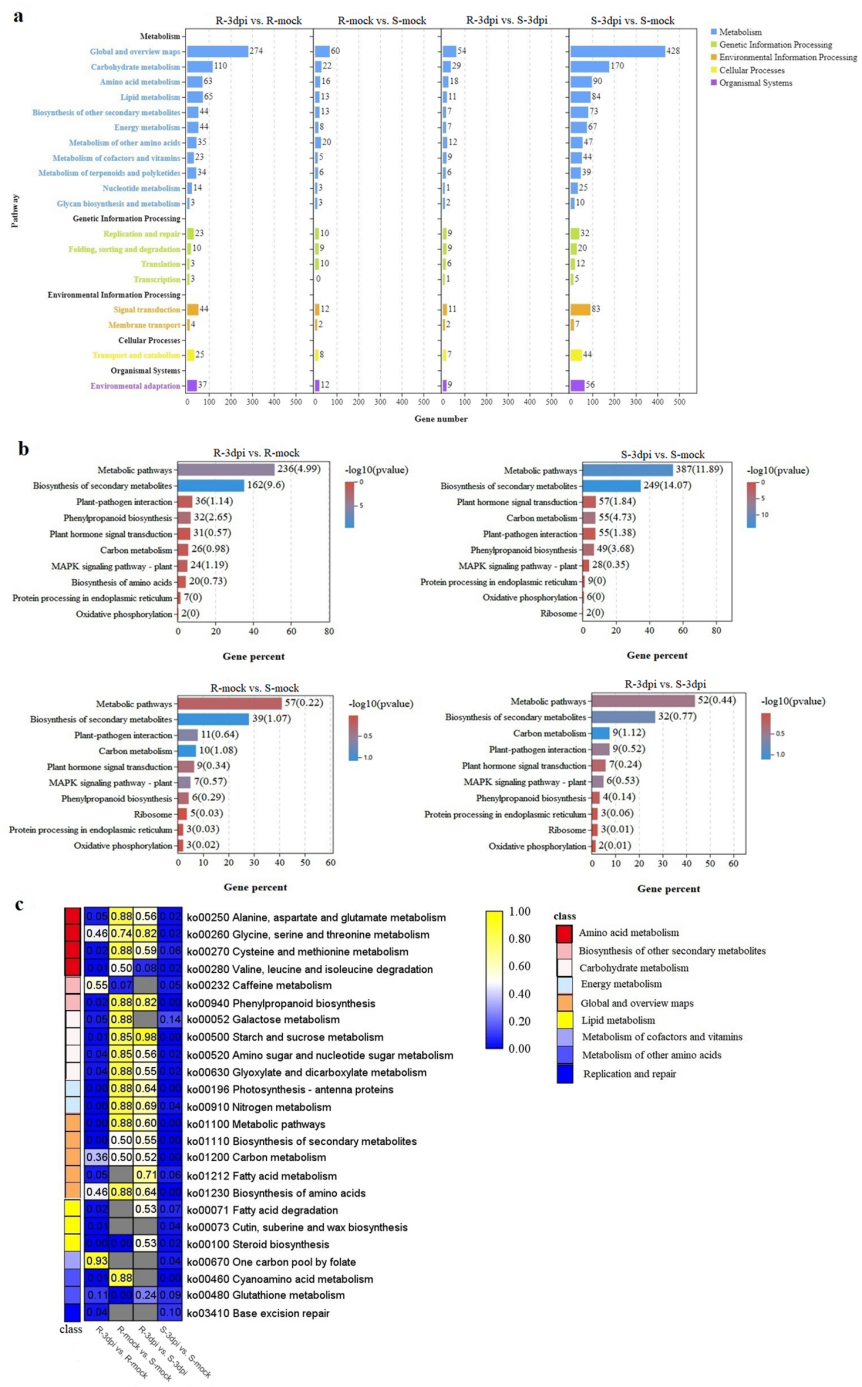
KEGG enrichment analysis of DEGs. (**a**) KEGG enrichment analyses in different comparisons. (**b**) The top ten KEGG pathways containing the largest number of DEGs in R-3dpi vs. R-mock, S-3dpi vs. S-mock, R-mock vs. S-mock, and R-3dpi vs. S-3dpi comparisons. (**c**) Q-value heat map of KEGG enrichment. R-mock represents mock-inoculated Hm 2–2 plants; R-3dpi represents 3 days post-pathogen-inoculated Hm 2–2 plants; S-mock represents mock-inoculated BY 1–2 plants; S-3dpi represents 3 days post-pathogen-inoculated BY 1–2 plants.

It is known that genes do not perform independently in any organism. They often coordinate and interact with each other to jointly regulate various life activities of the organism. The KEGG enrichment analysis was used to investigate the major pathways that the DEGs participated in. A total of 114, 124, 85, and 89 regulated (or altered) pathways in R-3dpi vs. R-mock, S-3dpi vs. S-mock, R-mock vs. S-mock, and R-3dpi vs. S-3dpi were identified, respectively (Fig. 6a and Supplementary Table S5–S8). The largest numbers of DEGs belonging to the top 10 KEGG pathways are shown in Fig. 6b. For R-3dpi vs. R-mock, more DEGs were enriched in the “Metabolic pathways” (236 DEGs), “Biosynthesis of secondary metabolites” (162 DEGs), “Plant-pathogen interaction” (36 DEGs), “Phenylpropanoid biosynthesis” (32 DEGs) and “Plant hormone signal transduction” (31 DEGs) pathways. Out of the 114 pathways, 24 had p-values < 0.05 (Supplementary Table S5). For S-3dpi vs. S-mock, most of the DEGs were enriched in “Metabolic pathways” (387 DEGs), followed by the “Biosynthesis of secondary metabolites” (249 DEGs), “Plant hormone signal transduction” (57 DEGs), “Carbon metabolism” (55 DEGs), and “Plant-pathogen interaction” (55 DEGs) pathways. On the other hand, out of the 124 pathways, 42 had p-values < 0.05 (Supplementary Table S6). In R-mock vs. S-mock, “Metabolic pathways” (57 DEGs), “Biosynthesis of secondary metabolites” (39 DEGs), “Plant-pathogen interaction” (11 DEGs), “Carbon metabolism” (10 DEGs), and “Plant hormone signal transduction” (9 DEGs) pathways showed the greatest enrichment. However, only 11 pathways had p-values < 0.05 (Supplementary Table S7). In R-3dpi vs. S-3dpi, “Metabolic pathways” (52 DEGs), “Biosynthesis of secondary metabolites” (32 DEGs), “Carbon metabolism” (9 DEGs), “Plant-pathogen interaction” (9 DEGs), and “Plant hormone signal transduction” (7 DEGs) showed the greatest enrichment; nine pathways had p-values < 0.05 (Supplementary Table S8). In addition, the Q-value of the KEGG enrichment analysis indicated that the largest number of enriched DEGs were involved in the biosynthesis of secondary metabolites, although the degree of enrichment might not be the highest compared with the other top enriched pathways (Fig. 6c).

### Plant–pathogen interaction pathways

Previous studies have concluded that increased Ca^2+^-dependent cyclic nucleotide-gated channels (CNGCs) play a key role in plant response to pathogens and pathogen-associated molecular pattern (PAMP) signals^51^. As shown in Supplementary Fig. S2a, the expression levels of CNGCs and HSP90 were up-regulated in R-mock vs. S-mock, but after *R. solanacearum* inoculation, their expression levels were significantly down-regulated (Supplementary Fig. S2b) in R-3dpi vs. S-3dpi. Furthermore, the CERK, MEKK1, Rboh, and PBS1 genes were down-regulated, but MYC2, PR1, and RIN4 were up-regulated in R-3dpi vs. S-3dpi. These results can help understand the resistance mechanism of tomato to *R. solanacearum*.

### Plant hormone signal transduction pathways

Most of the plant hormone signal transduction pathways were represented by DEGs in both genotypes with only a few differences. Auxin responsive *AUX/IAA* and *SAUR* were found up-regulated in both R-mock vs. S-mock and R-3dpi vs. S-3dpi. Cytokine responsive *B-ARR* was found up-regulated in R-mock vs. S-mock but not significantly changed in R-3dpi vs. S-3dpi comparison. Moreover, *AHP* was found down-regulated in R-3dpi vs. S-3dpi but no significant change in *AHP* expression was found in R-mock vs. S-mock. The TGA transcription factor, a regulator of NPR1, was found up-regulated in both R-mock vs. S-mock and R-3dpi vs. S-3dpi. Furthermore, the downstream *TGA* and *PR1* genes that are possibly responsible for disease resistance were up-regulated in both R-mock vs. S-mock and R-3dpi vs. S-3dpi (Supplementary Fig. S3).

### MAPK signaling pathways

A total of seven spots were mapped on the mitogen-activated protein kinase (MAPK) signaling pathway (Supplementary Fig. S4). *EBF1/2* genes in ethylene-responsive defense response were found down-regulated in both R-mock vs. S-mock and R-3dpi vs. S-3dpi comparisons, but interestingly, *ChiB* that was up-regulated in R-mock vs. S-mock showed down-regulation in R-3dpi vs. S-3dpi. Moreover, *EIN3/EIL* was found up-regulated in R-mock vs. S-mock but not significantly changed in R-3dpi vs. S-3dpi. Few plant-defense-related components, such as PR1 was found up-regulated, whereas *EBF1/2* were commonly down-regulated in both R-mock vs. S-mock and R-3dpi vs. S-3dpi. Similarly, *FLS2* was found down-regulated in both R-mock vs. S-mock and R-3dpi vs. S-3dpi. *MPKKK17/18* were found up-regulated in R-3dpi vs. S-3dpi but not significantly changed in R-mock vs. R-mock. Furthermore, cell death-related *RbohD* was found down-regulated in R-3dpi vs. S-3dpi but not significantly changed in R-mock vs. S-mock (Supplementary Fig. S4).

### Validation of RNA-seq data by qRT-PCR analysis

**Figure 7:**
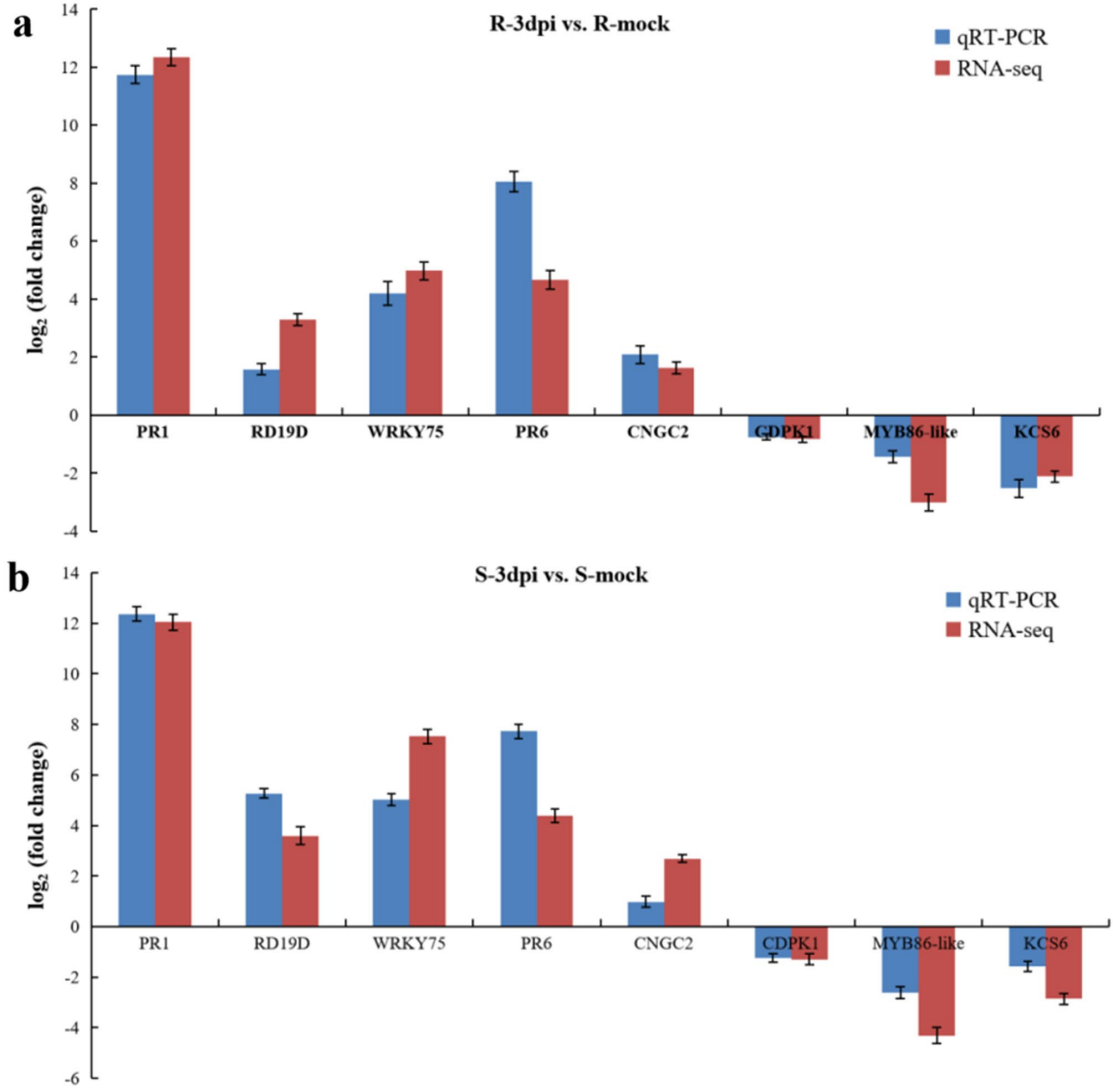
Quantitative real-time PCR validation. (**a**) In the R-3dpi vs. R-mock comparison. (**b**) In the S-3dpi vs. S-mock comparison. The red column represents RNA-seq data, and the blue column represents qRT-PCR data. The experiments were performed in triplicate. R-mock represents mock-inoculated Hm 2–2 plants; R-3dpi represents 3 days post-pathogen-inoculated Hm 2–2 plants; S-mock represents mock-inoculated BY 1–2 plants; S-3dpi represents 3 days post-pathogen-inoculated BY 1–2 plants.

To verify the reliability of Illumina sequencing results, eight genes were randomly selected for qRT-PCR analysis with three biological replicates. The expression levels of these genes were normalized to the constitutively expressed *Actin* gene. The genes including 3-ketoacyl-CoA synthase 6 (*KCS6*, Solyc05g009270.3), calcium-dependent protein kinase CDPK1 (*CDPK1*, Solyc07g064610.3), and transcription factor MYB86-like (*MYB86*, Solyc06g071690.3) were found down-regulated after *R. solanacearum* inoculation. But cysteine protease RD19D (*RD19D*, Solyc11g008260.2) gene, PR1 protein precursor (*PR1*, Solyc01g106620.2) gene, pathogenesis-related leaf protein 6 precursor (*PR6*, Solyc00g174340.2) gene, WRKY transcription factor 75 (*WRKY75*, Solyc05g015850.3) gene, and cyclic nucleotide-gated ion channel 2 isoform X2 (*CNGC2*, Solyc02g088560.3) gene were up-regulated after *R. solanacearum* inoculation. The verification result is shown in Fig. 7, and the primers for qRT-PCR are given in Supplementary Table S2. Overall, the results obtained from qRT-PCR and RNA-seq showed the same up- and down-regulation trends, which suggested that the RNA-seq results were reliable.

### Silencing of *WRKY75* in tomato leads to decreased resistance to *R. solanacearum*

**Figure 8:**
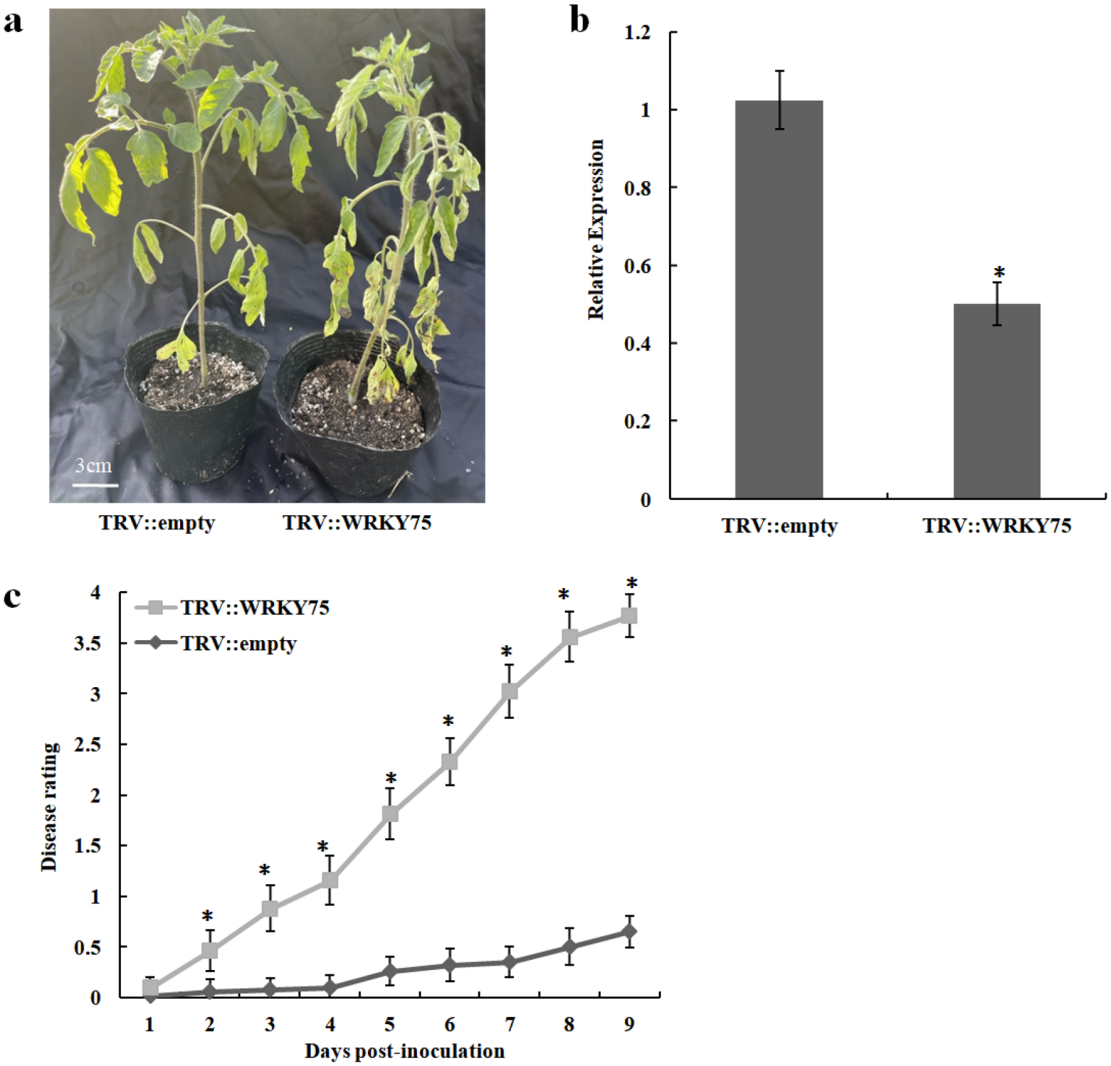
Resistance identification of tomato bacterial wilt after silencing *WRKY75* gene. (**a**) Phenotypic symtoms after inoculation with *Ralstonia solanacearum* of wild-type Hm 2–2 (TRV::empty) and silencing *WRKY75* (TRV::WRKY75) tomato plants; (**b**) qRT-PCR of *WRKY75* gene; (**c**) Disease scoring after infection with *R. solanacearum* in wild-type Hm 2–2 (dark gray) and silencing *WRKY75* (light gray) tomato plants. Results are averages ± s.e.m. (n = 20). *P < 0.05 using Student’s t-test. We repeated all experiments at least three times with similar results.

In order to further verify the reliability of the transcriptome data and the accuracy of qRT-PCR results, *WRKY75*, one of the eight DEGs was randomly selected for functional verification. Virus-induced gene silencing (VIGS) was used to verify the function of *WRKY75* gene. At 7 dpi, the resistant Hm 2–2 tomato plants inoculated with TRV::WRKY75 showed wilting symptoms, while plants inoculated with TRV::empty showed little wilting symptoms in their lower leaves, as shown in Fig. 8a. Figure 8b shows the expression pattern of *WRKY75* in VIGS plants. We evaluated the pathogenicity of the *WRKY75*-silencing plants in comparison with the wild-type Hm 2–2 plants (Fig. 8c). The result showed that the disease rating of *WRKY75-*silencing plants was greatly increased compared with the wild-type plants.

## Discussion

RNA-seq technology, namely “whole transcriptome shotgun sequencing” technology, is an effective method for comprehensive analysis of the entire transcriptome through high-throughput sequencing^52^. Compared with the conventional hybridization technique, RNA-seq technology, without advanced probe design, can test all transcription information for any species at the single nucleotide level, and it has gradually become a new technology of genome and transcriptome research due to its advantages including high-throughput, easy to operate, can be quantitative, and low cost. It is widely used in differential gene detection, new gene identification, and gene function analysis. As a next generation sequencing technology, RNA-seq technology has also achieved a great breakthrough in the study of plant disease resistance mechanisms. Strau et al.^53^ identified *Bs4C*, a candidate gene responsible for *Xanthomonas* resistance from pepper by RNA-seq technology. *Bs4C* can regulate the recognition of *Xanthomonas* transcription activator like effectors (TALE) Avr Bs4. To assess the susceptible response of apple to the fire blight pathogen *Erwinia amylovora*, Kamber et al.^54^ found that 1080 transcripts were differentially expressed at 48 h post-inoculation with *E. amylovora* through analyzing RNA-seq data from challenged and mock-inoculated flowers. Li et al.^55^ found 1196 and 358 defense-related genes were DEGs in resistant and susceptible plants, respectively, through deep RNA-seq analysis. Dasgupta et al.^56^ performed RNA-seq analysis between two genotypes containing PMR-1 (resistant) and Pusa Vishal (susceptible) mungbeans in response to yellow mosaic virus. The resistance to mungbean yellow mosaic India virus (MYMIV) showed a very complicated gene network, starting from the production of general PAMPs, and activating various signaling cascades such as brassinosteroid (BR), jasmonic acid (JA), and kinases. Finally, the expression of specific genes (such as R-gene proteins, virus resistance, and PR-proteins) leads to disease resistance response. In this study, we identified 1923 and 3274 DEGs in R-3dpi vs. R-mock and S-3dpi vs. S-mock comparisons, respectively, by using RNA-seq technology. The obtained results revealed that these DEGs were involved in plant metabolism, signal transduction, synthesis of secondary metabolites, genetic information processing, responses to environmental stimuli, self-immunity, and other life activities. These results showed that the quality of the transcriptome sequencing output and assembly that we obtained meets the requirements of transcriptome analysis. The use of RNA-seq technology can yield very comprehensive and abundant transcription information, and that can provide us with abundant resources and paths for future research.

Plant hormones are a class of organic substances produced by the plant itself. They play an important role in regulating various crucial activities including growth, development, senescence, and death at very low concentrations. Relevant studies have shown that SA, JA, and ET are the crucial signal molecules that induce plants to produce defense responses^57–59^. In addition, BRs, auxins, and cytokinins (CTKs) are also taking part in the defense response of plants to pathogens^60–62^. As a gaseous hormone, ET not only has a wide-ranging regulatory effect on plant growth and development but also participates in plant disease resistance and defense response^63–66^. Tezuka et al.^67^ reported that the rice ethylene response factor (ERF) OsERF83 positively regulates blast resistance by regulating the defense-related genes’ expression in rice. In this study, the expression of *EBF1/2* was up-regulated in the S-3dpi vs. S-mock comparison (Supplementary Fig. S3b). SA is a type of phenolic hormone widely distributed in plants. It participates in various physiological processes of plants and plays a key role in forming defense responses against pathogenic bacteria^68–70^. It has been proved that after plants are infected by pathogenic bacteria, the SA that increases exponentially in the body can induce plants to produce hypersensitive response (HR) and systemic acquired resistance (SAR) so that plants exhibit disease resistance^71^. The SA-mediated disease resistance signal transduction pathway is regulated by multiple genes. Among them, *NPR1* (*nonexpressor of PR-1*) is a key gene located downstream of the SA signal pathway. NPR1 interacts with the transcription factor TGA to activate the expression of a series of *SAR* genes, and finally confers improved resistance of plants to diseases^71^. Yang et al.^72^ reported that enhanced activation of SA biosynthesis in *Arabidopsis thaliana* hybrids may contribute to their increased resistance to a biotrophic bacterial pathogen. In this study, *NPR1* was down-regulated in both R-3dpi vs. R-mock and S-3dpi vs. S-mock comparisons, but *PR-1* was up-regulated only in the S-3dpi vs. S-mock (Supplementary Fig. S3). Moon et al.^73^ suggested that overexpression of *OsTGA2* can improve the resistance of rice against bacterial leaf blight disease by directly regulating the expression of defense-related genes. Liu et al.^74^ found that overexpression of *NtPR1a* contributed to increasing resistance to *R. solanacearum* in tobacco Yunyan 87 via activating the defense-related genes. Aux/IAAs (Auxin/indole-3-acetic acid proteins) play important roles in auxin signaling pathways, Fan et al.^75^ identified some MeAux/IAAs from *Manihot esculenta* as novel members in plant disease resistance through gene profiling and functional analysis, and these observations provide important information for further utilization of MeAux/IAAs. Similarly, AUX1 was found down-regulated after inoculation with pathogen (Supplementary Fig. S3). The plant hormone abscisic acid (ABA) was found in various parts of the plant. ABA can convert the adversity stimulus in the environment into internal signals of plant cells, and further, make plants to produce physiological reactions and resistance towards the invasion of pathogens^76,77^. Earlier studies have shown that the protein phospholipase 2 (*PP2C*) and sucrose non-glycolytic protein kinase 2 (*SnRK*) genes in plants can eliminate the inhibitory function of phosphate groups when the content of ABA is low, and then the production and release of ABA will be increased. It further promotes the formation of the ABA-PYR/PYL-PP2C complex, which in turn activates SnRK2. SnRK2 further activates the expression of downstream components, stress-related transcription factors, secondary messengers, and related genes, and regulates stress-responsive gene expression, ultimately protecting plants from adverse stresses^78–81^. In our study, *PP2C* and *SnRK2* were up-regulated in both R-3dpi vs. R-mock and S-3dpi vs. S-mock comparisons, but PYR/PYL was down- and up-regulated in R-3dpi vs. R-mock and S-3dpi vs. S-mock, respectively (Supplementary Fig. S3). In summary, several genes in the SA, auxin, ABA, and ET signaling pathways are involved in the defense response against *R. solanacearum* infection in tomato plants.

In the process of long-term interaction with pathogenic bacteria, plants have formed a set of the natural immune system, which includes two levels, namely PAMP-triggered immunity (PTI) and effector-triggered immunity (ETI). PTI promotes the recognition of PAMPs through cell surface receptors, such as bacterial flagellin and fungal chitin, thereby inducing host plants to produce a series of defense responses, including the formation of phytoalexins, the expression of disease-related proteins, and the deposition of the corpus callosum. ETI means that plants recognize the avirulence gene (*Avr*) of pathogens through the resistance gene (R), which triggers a series of specific defense responses^82–84^. In this study, Rd19 was up-regulated and CDPK, FLS2, and CERK were down-regulated in both R-3dpi vs. R-mock and S-3dpi vs. S-mock (Supplementary Fig. S2). Shi et al.^85^ reported that silencing of TaCP1 (an RD19-like cysteine protease) reduced wheat resistance to *Puccinia striiformis* f. sp. *tritici*. Fu et al.^86^ showed that rice *OsCPK10* is an important regulatory factor in plant immune response, which may regulate disease resistance by activating SA- and JA-dependent defense responses.

In addition, the study also found that the MAPK signal transduction pathway-related genes that mediate plants in response to pathogen infection are up-/down-regulated, respectively, including genes of MAPK and PR-1 (Supplementary Fig. S4). MAPK is a type of serine/threonine-protein kinase, which is ubiquitous in plants. It can receive extracellular or intracellular signals, and further integrate and amplify the signals, ultimately causing cell physiological responses. PR-1 is a pathogenesis-related protein (PRP) that is closely related to disease resistance in plants. This study has also been shown that various stimuli such as pathogen infection, mechanical damage, or pathogen elicitor can activate MAPK, and the MAPK system can further activate related transcription factors. The domains of transcription factors can combine with the W box (T) TGACC (A/T) of PR-1 and can quickly activate the early defense response signal of plants^87^. In this study, genes that displayed up-/down-regulated expression indicate that the MAPK signal transduction pathway is involved in the response of tomato to *R. solanacearum*.

## Conclusion

In this study, we analyzed the responses of BW-resistant and BW-susceptible tomato lines to *R. solanacearum* using RNA-seq. Gene expression changes following *R. solanacearum*-inoculation were identified, the expression levels and types of DEGs were investigated, and GO and KEGG enrichment analyses were performed to annotate the function of DEGs. Our results suggest complex and different responses of the two tomato inbred lines to *R. solanacearum* infection. This study provides an overall representation of the regulatory gene network for both resistant and susceptible tomato inbred lines responding to *R. solanacearum* infection. These *R. solanacearum*-responsive genes could serve as important candidates for future functional research and may be helpful in developing an effective method to resist this significant disease.

## Materials and methods

### Plant materials and growth conditions

Two *S. lycopersicum* inbred lines (selected and bred in our laboratory), Hm 2–2 (R, resistant to BW) and BY 1–2 (S, susceptible to BW), were used in this study. No approvals were required for this study, and we complied with all relevant regulations during the experiments. The collection, preservation, and use of plant materials involved in this study complied with relevant institutional, national, and international guidelines and legislation. *S. lycopersicum* seedlings (preserved in our laboratory) were planted in plastic trays (54 × 28 × 5 cm) filled with substrate (peat: perlite = 3:1, v/v).They were placed in an artificial climate chamber with a temperature of 28 ~ 30/15 ~ 17 °C (day/night), a photoperiod of 14/10 h (day/night), and relative humidity of 50% at Yichun University, Yichun, China. Three weeks later, 100 seedlings at the three-leaf stage were transplanted into black plastic pots (130 mm height × 150 mm diameter) filled with substrate (peat: perlite = 3:1, v/v), and plants were set 10–13 cm from each other. These plants were maintained in an artificial climate chamber with growth conditions as described above.

### Inoculation of *R. solanacearum*

The *R. solanacearum* strain belonging to race 1 biovar 3 was isolated from the BW-susceptible tomato line (BY 1–2)^88^. Bacteria were grown on triphenylterazolium chloride (TTC) medium (containing 3 g sucrose, 5–10 g tryptone, beef extract, and 7 g agar per liter) and incubated with 1% TTC for 2 days at 30 °C. Tomato plants at the six-leaf stage were inoculated with *R. solanacearum* by wounding the roots and incubating in the bacterial suspension (10^8^ colony forming units (cfu)/ml) for 30 min, while sterile water was used for mock-inoculation. Later, all inoculated plants were planted in the plastic pots. Pathogen-inoculated and mock-inoculated Hm 2–2 plants were denoted as R-3dpi and R-mock, respectively, while pathogen-inoculated and mock-inoculated BY 1–2 plants were denoted as S-3dpi and S-mock, respectively. Stem tissue samples of seven tomato plants at 3 dpi were flash-frozen in liquid nitrogen and stored at – 80 °C for further analysis. Each experiment was performed in triplicate.

### RNA extraction, RNA-seq library construction, and sequencing

Total RNA of the seven plant stem tissue samples from each of the four lines (R-mock, R-3dpi, S-mock, and S-3dpi) was extracted using a Trizol reagent kit (Invitrogen, Carlsbad, CA, USA) as per the manufacturer’s protocol. Each experiment was performed in triplicate. RNA quality was assessed on an Agilent 2100 Bioanalyzer (Agilent Technologies, Palo Alto, CA, USA) and checked using RNase-free agarose gel electrophoresis. After extracting the total RNA, eukaryotic mRNA was enriched by oligo (dT) beads, while prokaryotic mRNA was enriched by removing rRNA by Ribo-Zero Magnetic Kit (Epicentre, Madison, WI, USA). The enriched mRNA was fragmented into short fragments using fragmentation buffer and reverse transcribed into complementary DNA (cDNA) with random primers. Second-strand cDNA was synthesized by DNA polymerase I, RNase H, dNTP, and buffer. Then the cDNA fragments were purified with the QiaQuick PCR extraction kit (Qiagen, Venlo, the Netherlands), end-repaired, poly(A)-added, and ligated to Illumina sequencing adapters. The ligation products were size selected by agarose gel electrophoresis, PCR amplified, and sequenced using an Illumina HiSeq2500 platform by Gene Denovo Biotechnology Co. (Guangzhou, China).

### RNA-seq data analysis

Raw reads obtained from the sequencer contain adapters or low-quality bases, which would affect the following assembly and analysis. Thus, to get high-quality clean reads, raw reads were filtered by fastp v0.18.0^89^. Short reads alignment tool Bowtie2 v2.2.8 was used for mapping reads to the ribosome RNA (rRNA) database^90^. The rRNA-mapped reads were removed. The remaining clean reads were further used in assembly and gene abundance calculation. An index of the reference genome was built, and paired-end clean reads were mapped to the *S. lycopersicum* reference genome (ITAG3.2) using HISAT v2.2.4 with “-rna-strandness RF”, while other parameters were set as default^91^. The mapped reads of each sample were assembled by using a reference-based approach by StringTie v1.3.1^92,93^. For each transcription region, an FPKM (fragment per kilobase of transcript per million mapped reads) value was calculated to quantify its expression abundance and variations, using StringTie v1.3.1 software.

### Analysis of DEGs

DEGs were detected using the DESeq2 package^94^. The analysis mainly consisted of three steps: (1) normalization of the read count; (2) calculation of the probability of hypothesis testing (p-value) according to the model; (3) conduction of multiple hypothesis testing to obtain the false discovery rate (FDR) value. The genes with a p-value ≤ 0.05 and |log2FC|≥ 1 were considered DEGs.

### GO enrichment analysis

Gene ontology (GO) is an international standardized gene functional classification system that offers a dynamic-updated controlled vocabulary and a strictly defined concept to a comprehensive description of gene properties and their products in any organism^95^. GO has three ontologies: molecular function, cellular component, and biological process. The basic unit of GO is GO term. Each GO term belongs to a type of ontology. GO enrichment analysis provides all GO terms that are significantly enriched in DEGs comparing to the genome background and filter the DEGs that correspond to biological functions. First, all DEGs were mapped to the GO database and assigned with GO terms. Then, gene numbers were calculated for each GO term. Finally, significantly enriched GO terms among DEGs comparing to the genome background were defined by a hypergeometric test. This analysis is able to recognize the main biological functions that DEGs participate in.

### Pathway enrichment analysis

Genes usually interact with each other to play roles in certain biological processes. Pathway-based analysis helps to further understand gene biological functions. KEGG (Kyoto Encyclopedia of Genes and Genomes) is the major public pathway-related database^96,97^. The KEGG pathway was taken as the unit and a hypergeometric test was used to identify pathways that were significantly enriched among the DEGs compared to the entire genome background. Through significant enrichment of pathways, the most important biochemical metabolic pathways and signal transduction pathways involving DEGs can be determined.

### Single-nucleotide polymorphism (SNP) analysis

Variation detection based on transcriptome sequencing mainly includes SNPs and insertions/deletions (InDels). SNP variation refers to the DNA sequence polymorphism caused by the change of a single nucleotide at the genome level, while InDel is the variation caused by the insertion or deletion of a nucleotide. The software GATK v3.4-6 was used to detect the variation of SNPs and InDels and finally conduct data statistics^98^.

### Validation of RNA-seq data by qRT-PCR

To validate the RNA-seq results, quantitative real-time PCR (qRT-PCR) was performed in this study. RNA was extracted using the HiPure Total RNA Mini Kit (Magen, Guangzhou, China). Later, RNA was reverse transcribed to cDNA using the HiScript II 1st Stand cDNA Synthesis Kit (+ gDNA wiper; Vazyme, Nanjing, China). The qRT-PCR was performed in triplicate for each sample using the 2 × RealStar Green Fast Mixture (with ROX) as per the manufacturer’s instruction manual. The qRT-PCR amplification was performed using the StepOne Real-Time PCR Instrument (Applied Biosystems, Thermo Fisher, USA) and corresponding software (Applied Biosystems). The tomato *Actin* (Solyc03g078400) gene was used as the internal control^99^. The relative expression levels of the genes from three biological replicates were calculated using the 2^−△△Ct^ method^100^. All primers for qRT-PCR are listed in Supplementary Table S1.

### Vector construction and virus-induced gene silencing (VIGS) transformation

**Figure 9:**
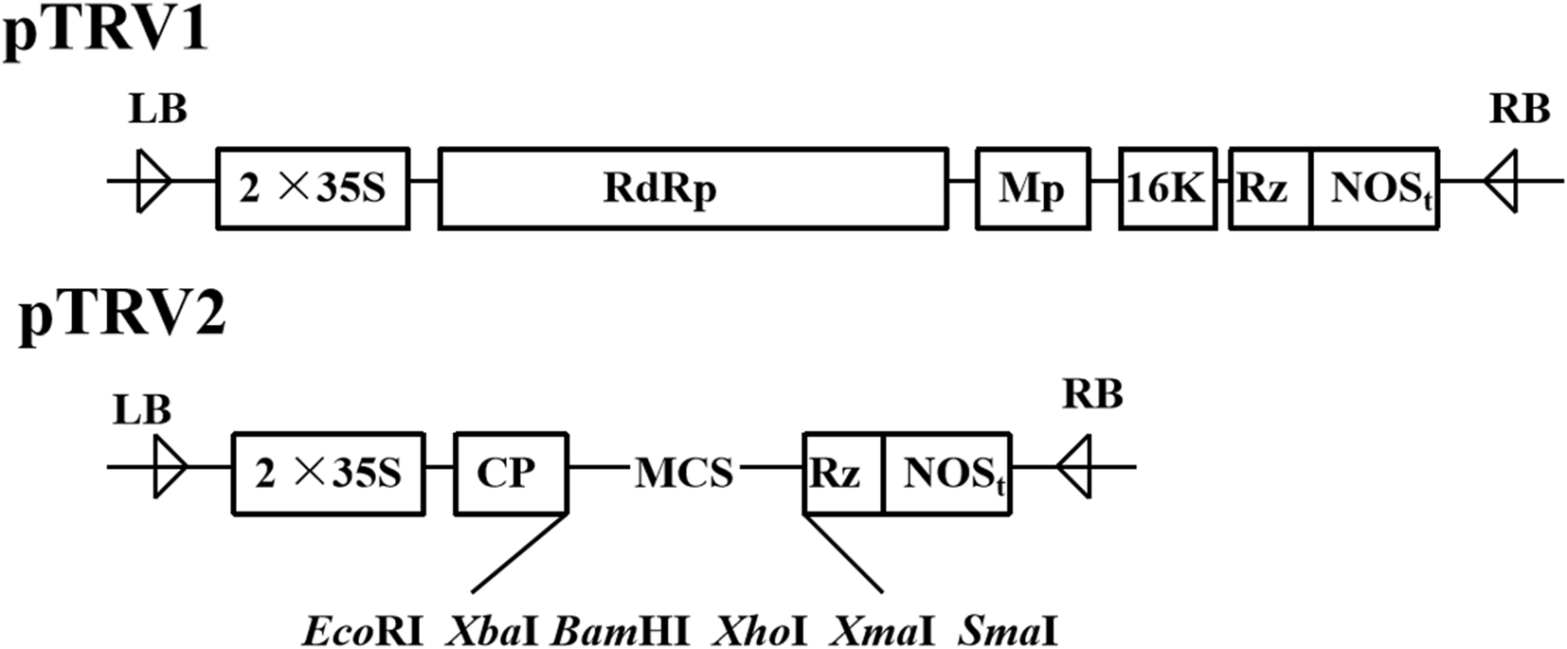
The map of pTRV1 and pTRV2 vectors.

The VIGS vectors pTRV1 and pTRV2^101^ were stored in our laboratory (Fig. 9). A 199-bp *WRKY75* (initially characterized by López-Galiano et al.^102^ and Chen et al.^103^) fragment was amplified from the stem tissue of resistant tomato plants (Hm 2–2) with the specific primers V-F and V-R (listed in Supplementary Table S2) by PCR. The *WRKY75* fragment and the vector pTRV2 were then digested with *EcoR* I and *BamH* I (Takara, China), and the *WRKY75* fragment was ligated into the vector using T4 ligase (Takara, China) to create the TPV::WRKY75 construct; pTRV2 with empty was created to TRV::empty and was used as control. The resulting vector was introduced into *Agrobacterium tumefaciens* GV3101 (WEIDI, China). The VIGS experiments were carried out as described previously^104^. Three-week-old newly emerged leaves of Hm 2–2 tomato plants were transformed with *Agrobacterium* containing TPV::WRKY75 (V(pTRV1):V(pTRV2::WRKY75) = 1:1) and TRV::empty (V(pTRV1):V(pTRV2::empty) = 1:1) vectors, respectively, using a 1-mL syringe. Each experiment included three biological replicates. One week later, the plants were inoculated with *R. solanacearum* by using the root-cutting and root-grafting method. Disease scoring was performed according to that previously described by Lacombe et al^105^.

### Statistical analysis

Data were expressed as the mean ± standard deviation (SD). Statistical analyses were performed by SPSS (Statistical Product and Service Solutions) and Excel 2007. Differences were considered statistically significant at p < 0.05 and p < 0.01.

## Supplementary Information

Supplementary Information 1.

Supplementary Information 2.

Supplementary Information 3.

Supplementary Information 4.

Supplementary Information 5.

## Data Availability

RNA sequencing data were deposited at the National Center for Biotechnology Information (NCBI) in the Sequence Read Archive (SRA) under the PRJNA787007 Bioproject accession.
