# Supplementary material for: Transcriptome analysis reveals differential transcription in tomato (Solanum lycopersicum) following inoculation with Ralstonia solanacearum
Source: Sci Rep. 2022 Dec 22;12:22137. doi: 10.1038/s41598-022-26693-y (PMC9780229; doi:10.1038/s41598-022-26693-y)
Supplement: Supplementary file 1 — Supplementary Information 1. [file 41598_2022_26693_MOESM1_ESM.doc]

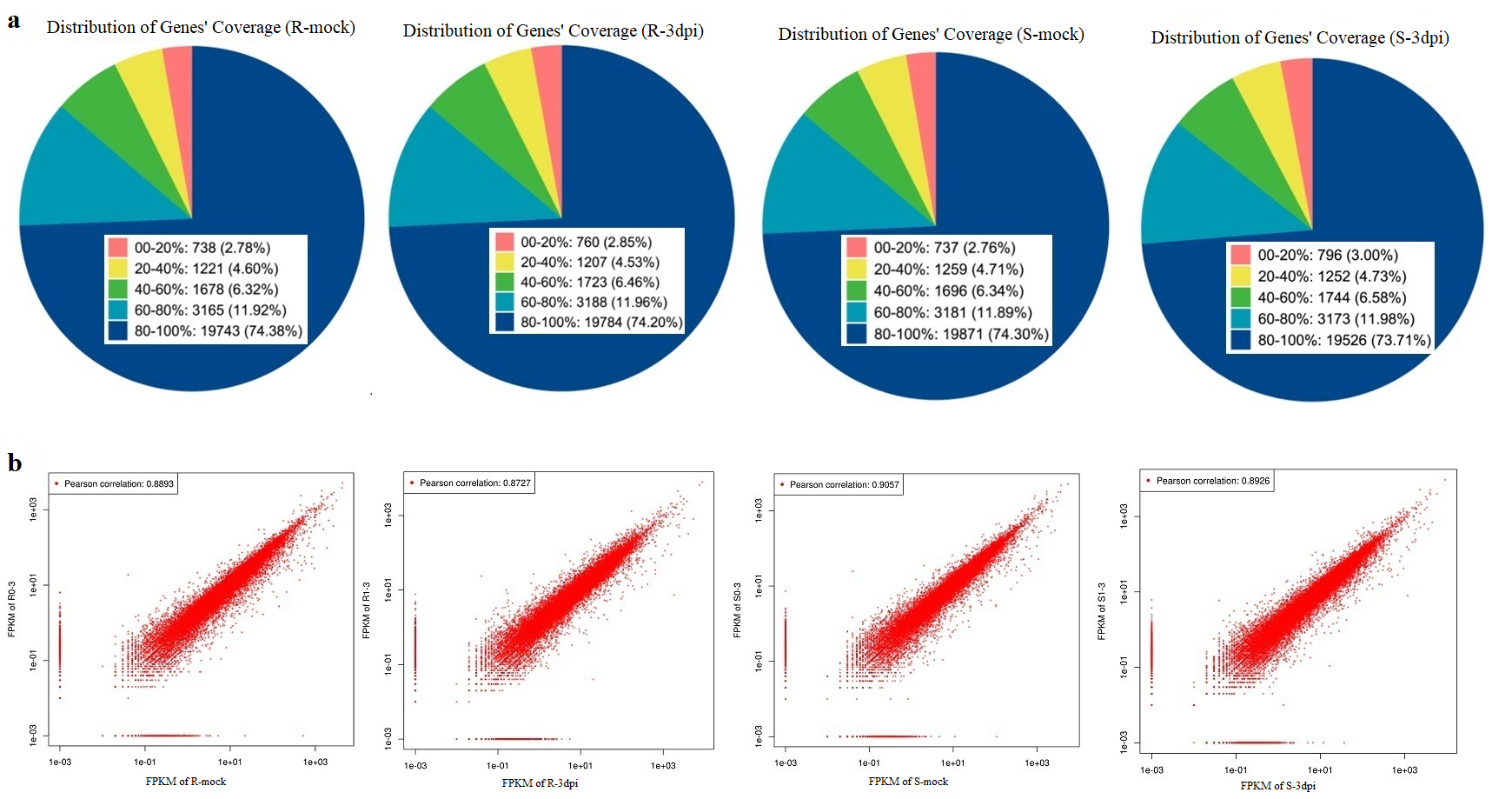


**Figure S1.** Transcriptome sequencing quality analysis between treatments and control. **a:** Distribution of genes’ coverage; **b:** Correlation coefficient maps between different sequencing replicates. R-mock represents mock-inoculated Hm 2-2 plants; R-3dpi represents 3 days post-pathogen-inoculated Hm 2-2 plants; S-mock represents mock-inoculated BY 1-2 plants; S-3dpi represents 3 days post-pathogen-inoculated BY 1-2 plants.


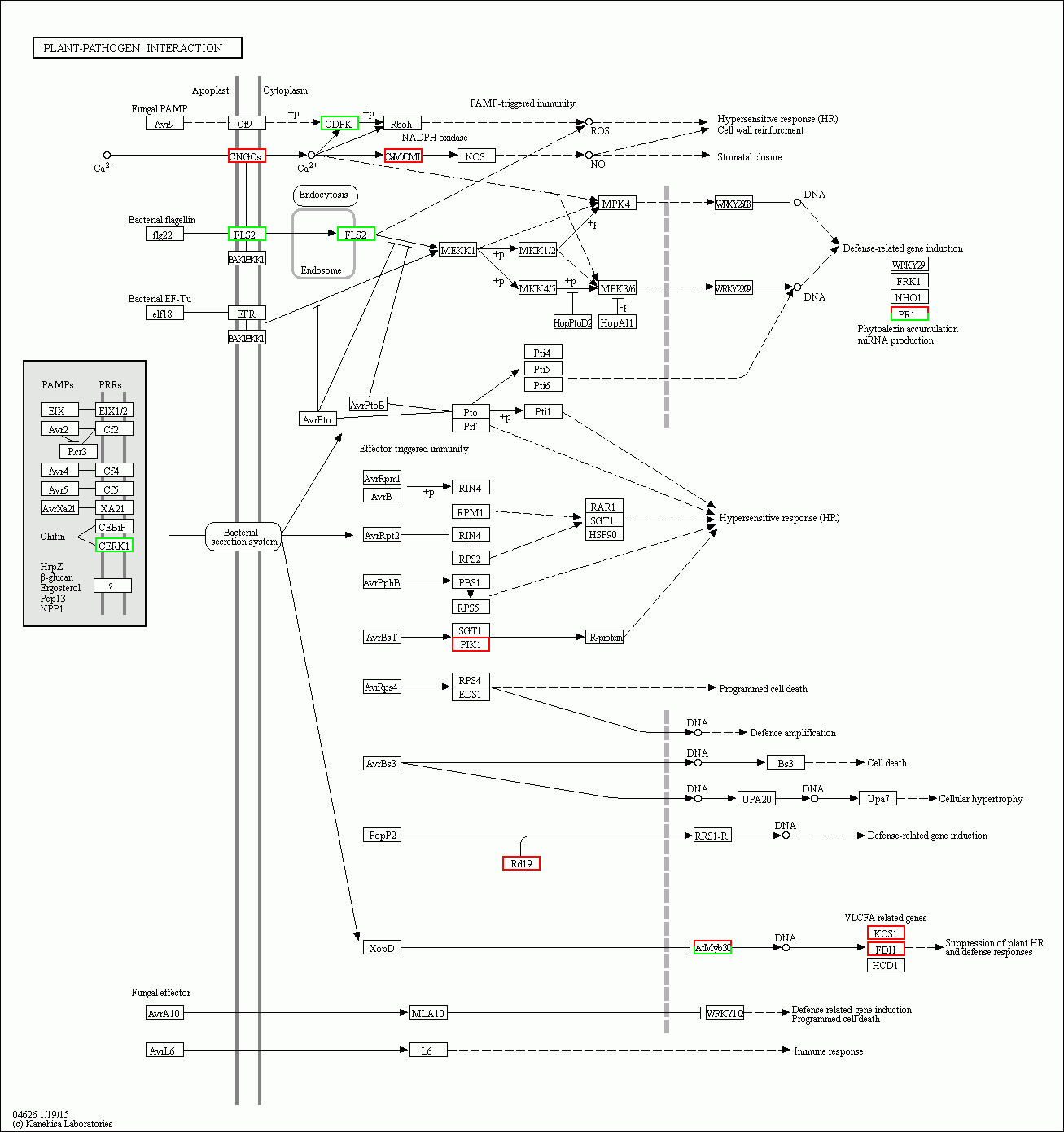


**a**


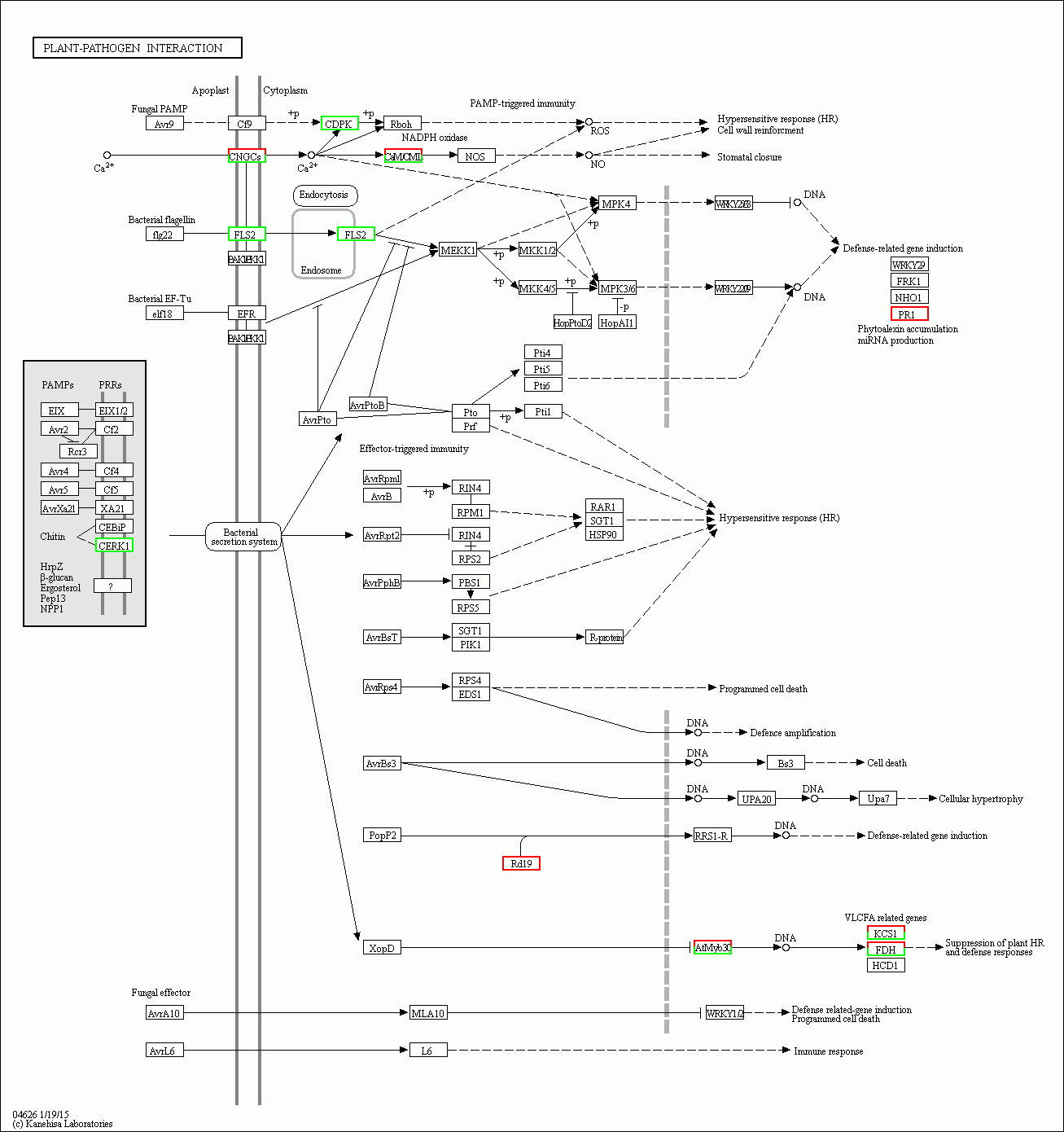


**b**

**Figure S2.** Plant-pathogen interaction pathway. **a**: In comparison of R-3dpi vs. R-mock. R-mock represent mock-inoculated Hm 2-2 plants, and R-3dpi represent 3 days post-pathogen-inoculated Hm 2-2 plants; **b**: In comparison of S-3dpi vs. S-mock. S-mock represent mock-inoculated BY 1-2 plants, and S-3dpi represent 3 days post-pathogen-inoculated BY 1-2 plants. Red borders indicate up-regulated genes, green borders indicate down-regulated genes and black borders indicate non-change genes.


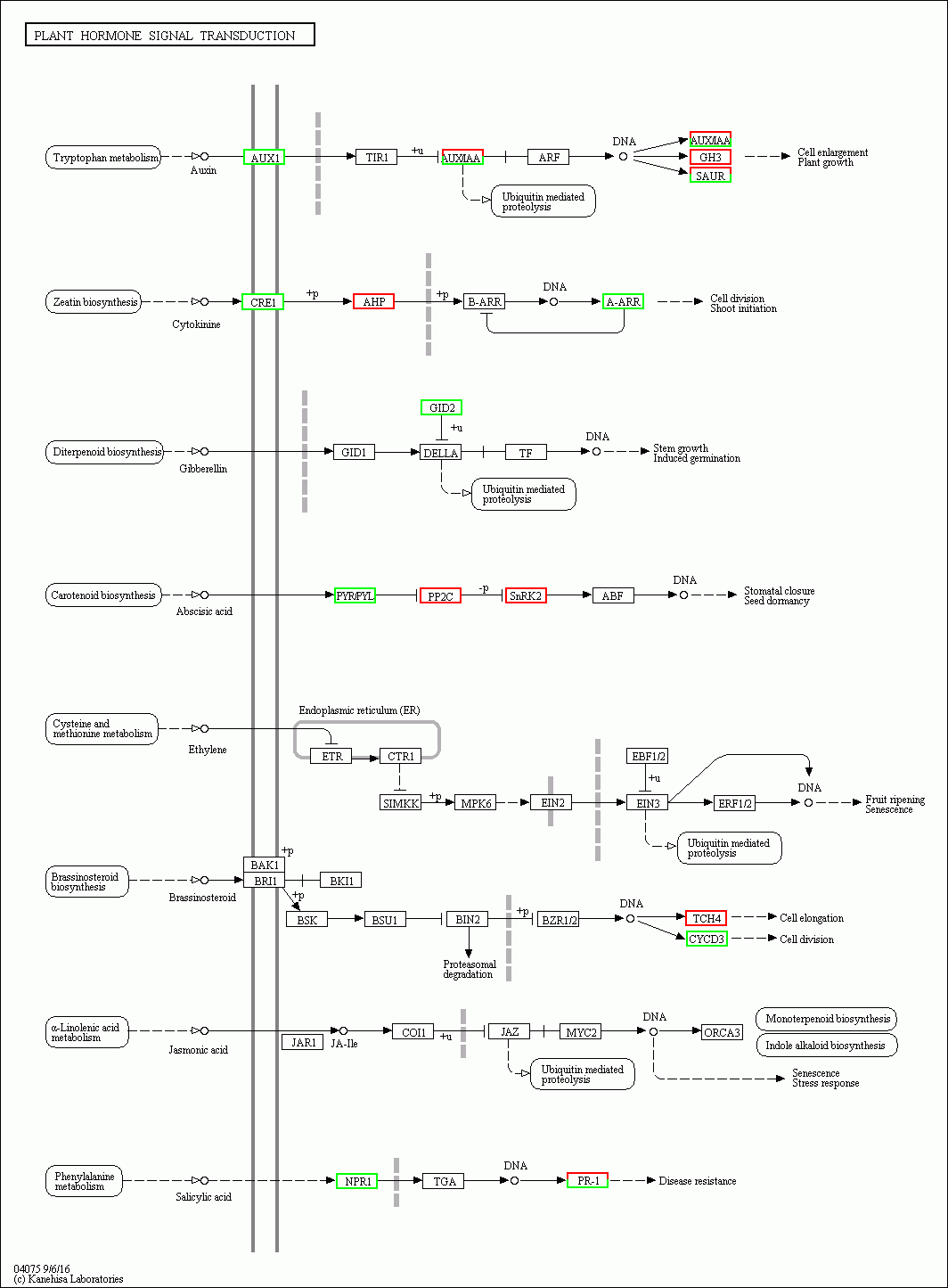


**a**


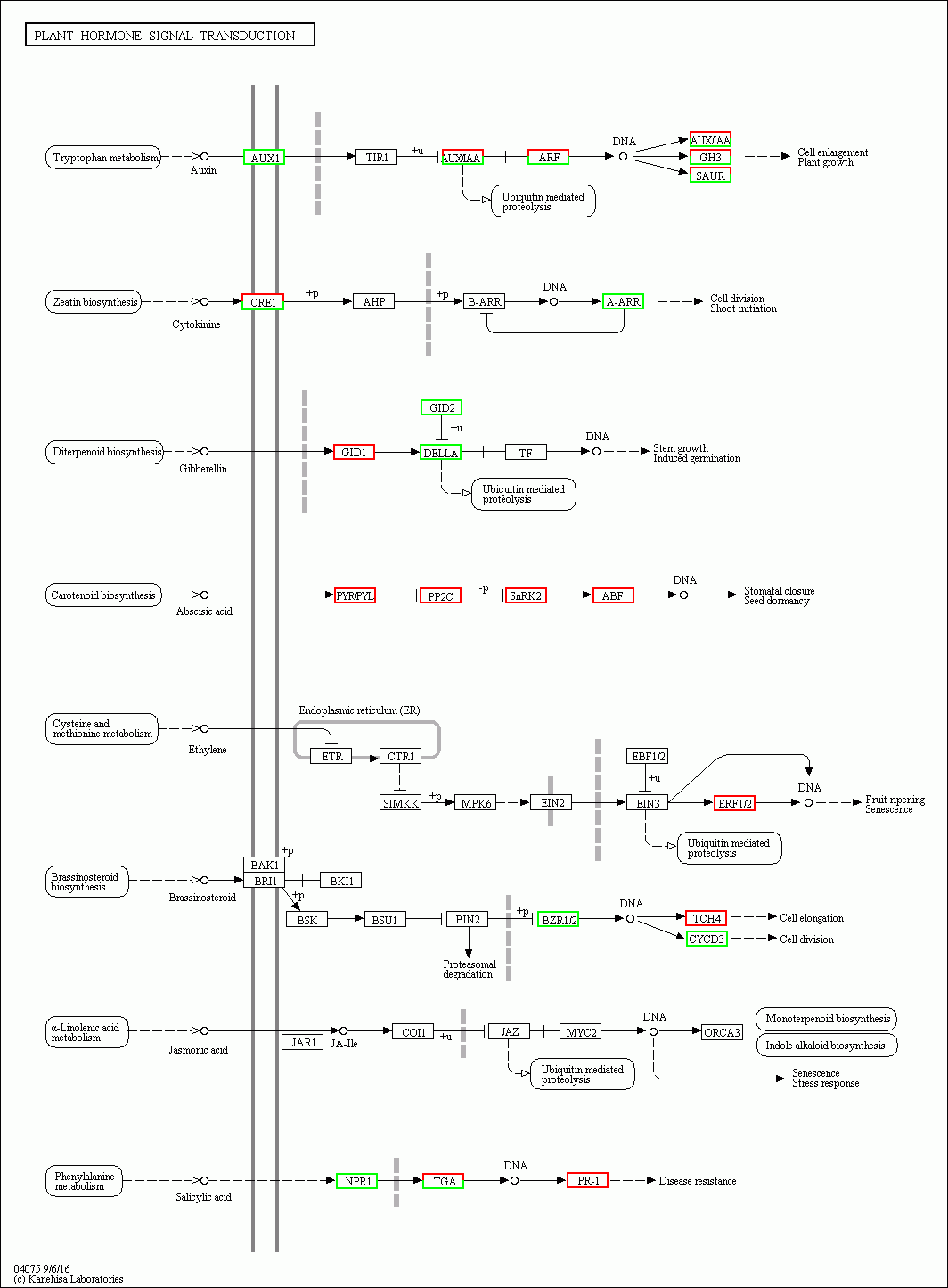


**b**

**Figure S3.** Plant hormone signal transduction pathway. **a**: In comparison of R-3dpi vs. R-mock. R-mock represent mock-inoculated Hm 2-2 plants, and R-3dpi represent 3 days post-pathogen-inoculated Hm 2-2 plants; **b**: In comparison of S-3dpi vs. S-mock. S-mock represent mock-inoculated BY 1-2 plants, and S-3dpi represent 3 days post-pathogen-inoculated BY 1-2 plants. Red borders indicate up-regulated genes, green borders indicate down-regulated genes and black borders indicate non-change genes.


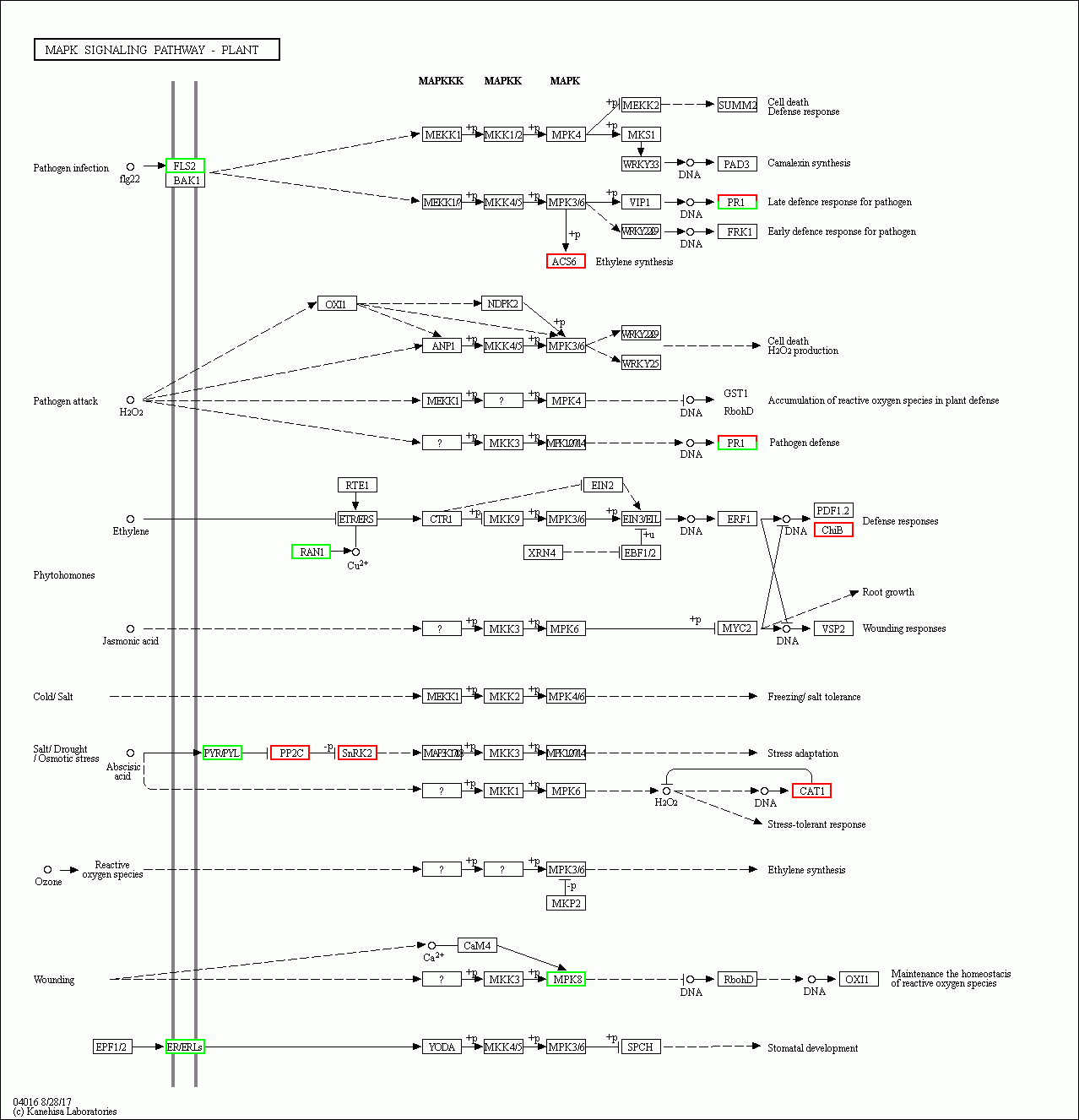


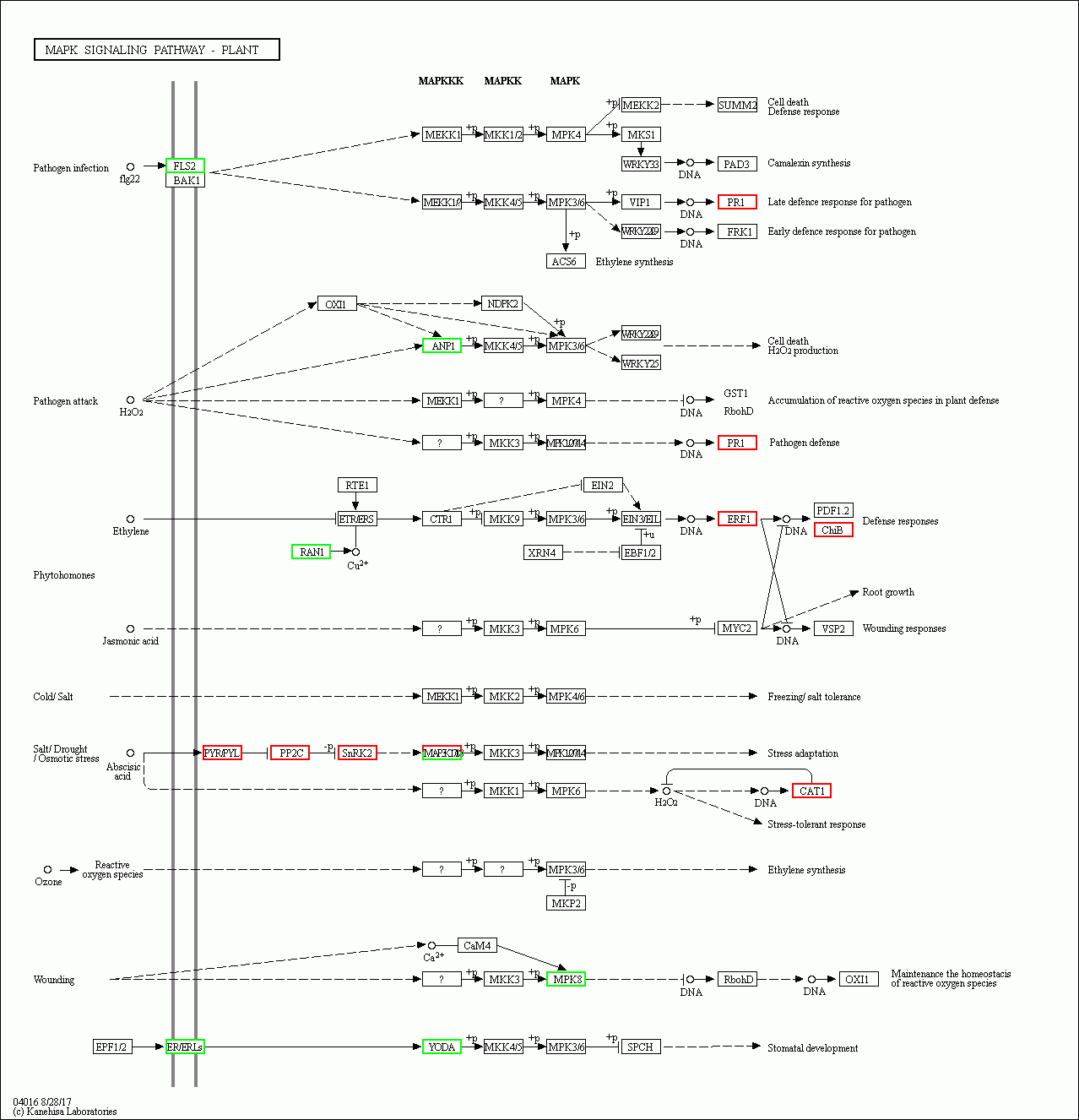


**b**

**Figure S4.** MAPK signaling pathway. **a**: In comparison of R-3dpi vs. R-mock. R-mock represent mock-inoculated Hm 2-2 plants, and R-3dpi represent 3 days post-pathogen-inoculated Hm 2-2 plants; **b**: In comparison of S-3dpi vs. S-mock. S-mock represent mock-inoculated BY 1-2 plants, and S-3dpi represent 3 days post-pathogen-inoculated BY 1-2 plants. Red borders indicate up-regulated genes, green borders indicate down-regulated genes and black borders indicate non-change genes.

| **Group** | **Sample Name** | **Raw Reads** | **Clean Reads** | **Clean Reads Rate (%)** | **Mapped Reads (%)** | **Uniquely Reads (%)** | **Q20 (%)** |
| --- | --- | --- | --- | --- | --- | --- | --- |
| 1 | R-mock (Rep 1) | 36,162,686 | 36,066,666 | 99.73% | 96.77% | 95.22% | 97.71% |
|  | R-mock (Rep 2) | 32,255,930 | 32,179,868 | 99.76% | 96.37% | 94.81% | 97.81% |
|  | R-mock (Rep 3) | 36,317,478 | 36,229,616 | 99.76% | 97.07% | 95.53% | 97.92% |
| 2 | R-3dpi (Rep 1) | 33,492,004 | 33,398,948 | 99.72% | 96.82% | 95.28% | 97.76% |
|  | R-3dpi (Rep 2) | 36,640,430 | 36,535,498 | 99.71% | 96.31% | 94.73% | 97.75% |
|  | R-3dpi (Rep 3) | 36,774,446 | 36,665,492 | 99.70% | 96.91% | 95.36% | 97.95% |
| 3 | S-mock (Rep 1) | 34,772,294 | 34,683,264 | 99.74% | 97.52% | 95.89% | 97.88% |
|  | S-mock (Rep 2) | 36,034,642 | 35,952,148 | 99.77% | 97.71% | 96.09% | 97.94% |
|  | S-mock (Rep 3) | 36,498,362 | 36,392,352 | 99.71% | 97.43% | 95.85% | 98.02% |
| 4 | S-3dpi (Rep 1) | 37,394,100 | 37,280,266 | 99.70% | 96.73% | 95.21% | 97.33% |
|  | S-3dpi (Rep 2) | 36,630,926 | 36,528,250 | 99.72% | 97.60% | 96.00% | 97.96% |
|  | S-3dpi (Rep 3) | 36,324,596 | 36,231,856 | 99.74% | 97.49% | 95.80% | 97.87% |

**Table S1.** Summary statistics of RNA-Seq data and mapping results.

Note: Group 1, and 2 comprise of mock-inoculation and pathogen-inoculation Hm 2-2 plants, group 3 and 4 comprise of mock-inoculation and pathogen-inoculation BY 1-2 plants.

| **Gene ID** | **Gene Name** | **Primers(5’-3’)** |
| --- | --- | --- |
| Solyc05g009270.3 | KCS6 | F: ATCATCGTCGTCACTATGGTAC  R: CTGTCAATACAATCAGCCCATG |
| Solyc07g064610.3 | CDPK1 | F: GATTACTGATTTTGGGCTGTCG  R: CTTGCCATAACACCTCCTTAGA |
| Solyc06g071690.3 | MYB86-Like | F: CAAATTGCAGCAAAATTACCGG  R: CTTAGTGGCTTGTGGGTATTTG |
| Solyc11g008260.2 | RD19D | F: GAGAGAGAAAGGTGCTGTTACT  R: AGTTGGCTCCTTCAATAGATCC |
| Solyc01g106620.2 | PR1 | F: GGCTGGTGCTGTGAAGAT  R: CCTGACCCTAGCACAACC |
| Solyc00g174340.2 | PR6 | F: TGCCAAGGGTGGTGGTGAC  R: CGTGCCCGACCACAACCTA |
| Solyc05g015850.3 | WRKY75 | F: GTAGTTACTACTTATGAAGGCATGC  R: GCATTTGACTCAAAATGTGCTC |
| Solyc02g088560.3 | CNGC2 | F: ACCTGGGCTGCTGTGAAT  R: ATACTTGCGAAGACGATG |
| Solyc03g078400 | SlACTIN | F: CTCTACATACTTGAGAGGTGCC  R: AGACGAGGAGAAAACATCACAA |
| Solyc05g015850.3 | WRKY75 | V-F:CCGGAATTCTTTCTTGGGACTAATGGC  V-R: CGCGGATCCCCTTGGGAATTTGTTGTT |

**Table S2.** Primers used for in this study.

| **Gene ID** | **Symbol** | **Log_2_ (fold change)** | | | | **Description** |
| --- | --- | --- | --- | --- | --- | --- |
|  |  | **R-3dpi vs. R-mock** | **S-3dpi vs. S-mock** | **R-mock vs. S-mock** | **R-3dpi vs. S-3dpi** |  |
| Solyc05g005540.3 | GP3 | -4.51 | -2.63 | 0.63 | 2.52 | polygalacturonase non-catalytic subunit AroGP3 precursor |
| Solyc09g014530.3 | MLP168 | -3.28 | -3.52 | 1.69 | 1.45 | kirola-like |
| Solyc09g084450.3 | PIIF | -4.36 | -4.07 | -1.87 | -1.59 | wound-induced proteinase inhibitor 1-like |
| Solyc12g040790.2 | NMT2 | -5.22 | -6.57 | -0.08 | -1.43 | phosphomethylethanolamine N-methyltransferase |
| Solyc11g020960.2 | PIN2 | -3.63 | -4.01 | -1.35 | -1.73 | wound-induced proteinase inhibitor 2-like |
| Solyc12g013710.2 | POR1 | -3.28 | -4.41 | -0.48 | -1.61 | protochlorophyllidereductase-like |
| Solyc12g044950.2 | FAD2 | -2.62 | -4.71 | 0.05 | -2.045 | lipid desaturase-like protein |
| Solyc06g069730.3 | LHCA4 | -4.01 | -5.22 | 0.35 | -0.86 | chlorophyll a-b binding protein Cab12 |
| Solyc08g078900.1 | EARLI1 | -3.78 | -4.87 | 0.03 | -1.05 | arachidonic acid-induced DEA1 precursor |
| Solyc09g008860.3 | PXC1 | -3.37 | -4.51 | 0.12 | -1.03 | leucine-rich repeat receptor-like protein kinase PXC1 |
| Solyc01g096450.3 | AED3 | -2.85 | -3.48 | -0.30 | -0.92 | aspartyl protease AED3 |
| Solyc07g045440.1 | FLA2 | -2.77 | -3.39 | -0.08 | -0.70 | fasciclin-like arabinogalactan protein 2 |
| Solyc03g031730.3 | BGLU47 | -3.29 | -3.40 | 0.42 | -0.29 | beta-glucosidase 46 |
| Solyc11g013810.2 | NIA | -3.40 | -4.24 | 0.72 | -0.12 | nitrate reductase [NADH] |
| Solyc02g068877.1 | EXL7 | -4.03 | -3.92 | -0.57 | -0.47 | protein EXORDIUM-like 7 |
| Solyc06g062380.3 | APS1 | -4.08 | -4.21 | -0.52 | -0.65 | acid phosphatase 1 |
| Solyc02g065530.3 | GATL1 | -3.85 | -3.77 | -0.11 | -0.03 | probable galacturonosyltransferase-like 1 |
| Solyc04g014510.3 | GLN1-1 | -3.94 | -4.49 | 0.28 | -0.28 | glutamine synthetase cytosolic isozyme 1-1 |
| Solyc12g009270.1 | PMEI3 | -4.10 | -4.34 | -0.07 | -0.31 | 21 kDa protein-like |
| Solyc03g005940.3 | OBAP2A | 13.25 | 9.12 | 4.74 | 0.61 | oil body-associated protein 2A-like |
| Solyc04g079047.1 | SCPL10 | 12.95 | 7.27 | 5.82 | 0.14 | serine carboxypeptidase-like 13 |
| Solyc01g106620.2 | PR1B1 | 12.35 | 12.05 | 0.49 | 0.19 | PR1 protein precursor |
| Solyc04g072250.3 | HSP18 | 9.53 | 8.98 | 0 | -0.55 | 16.9 kDa class I heat shock protein 1-like |
| Solyc09g082340.2 | AMP2-1 | 10.14 | 8.87 | -0.58 | -1.85 | vicilin precursor |
| Solyc03g044460.1 | HEC2 | 7.68 | 13.45 | -5.82 | -0.06 | transcription factor HEC2-like |
| Solyc07g007710.3 | SD2 | 7.42 | 12.69 | -6.13 | -0.87 | defensin-like protein precursor |
| Solyc02g076910.3 | SAG12 | 5.14 | 8.57 | -2.21 | 1.21 | senescence-specific cysteine proteaseSAG39-like |
| Solyc05g015850.3 | WRKY75 | 4.98 | 7.53 | -2.01 | 0.54 | probable WRKY transcription factor 75 |
| Solyc02g071430.3 | SRG1 | 7.49 | 7.98 | -0.46 | 0.037 | protein SRG1 |
| Solyc06g072130.3 | TIP3-2 | 7.14 | 7.98 | -1.20 | -0.35 | probable aquaporin TIP3-2 |
| Solyc11g044910.2 | BXL1 | 6.86 | 8.56 | -0.09 | 1.61 | SlArf/Xyl1 protein precursor |
| Solyc10g078770.2 | LE25 | 7.81 | 7.91 | 0.50 | 0.60 | protein LE25 |
| Solyc03g019820.3 | TIP3-2 | 8.41 | 8.25 | 0.69 | 0.52 | probable aquaporin TIP3-2-like |
| Solyc03g025810.3 | LTI65 | 8.58 | 8.22 | 0.62 | 0.26 | low-temperature-induced 65 kDa protein-like |
| Solyc12g011310.2 | PARC | 3.23 | 4.70 | -1.97 | -0.50 | probable glutathione S-transferaseparC |
| Solyc06g060970.2 | EXLB1 | 4.33 | 4.41 | -1.01 | -0.92 | expansin-like B1 |
| Solyc02g084850.3 | TAS14 | 3.94 | 3.58 | -0.42 | -0.77 | abscisic acid and environmental stress-inducible protein TAS14 |
| Solyc04g007630.2 | HIPP20 | 4.84 | 3.81 | -0.07 | -1.10 | heavy metal-associated isoprenylated plant protein 20-like |
| Solyc01g097270.3 | WIN2 | 4.51 | 3.33 | 1.09 | -0.09 | wound-induced protein WIN1-like |
| Solyc10g055800.2 | CHTB3 | 4.13 | 3.33 | 0.70 | -0.11 | endochitinase 4 |
| Solyc00g174340.2 | PR1B1 | 4.66 | 4.38 | 0.88 | 0.60 | pathogenesis-related leaf protein 6 precursor |
| Solyc08g080650.3 | TPM-1 | 5.15 | 3.92 | 1.33 | 0.10 | osmotin-like protein TPM-1 precursor |
| Solyc04g005610.3 | NAC029 | 4.36 | 5.68 | -0.20 | 1.12 | NAC transcription factor 29 |
| Solyc03g098100.3 | COR2 | 5.03 | 5.62 | 0.02 | 0.61 | methylecgononereductase |
| Solyc01g067300.3 | IN26 | 4.46 | 5.23 | 0.05 | 0.82 | CASP-like protein 1 |
| Solyc10g083690.3 | CYP76A2 | 4.79 | 5.27 | 0.44 | 0.93 | cytochrome P450 76A2-like |
| Solyc01g079300.3 | STS1 | 5.63 | 5.26 | 0.24 | -0.13 | stachyose synthase |
| Solyc12g008430.2 | ME1 | 6.10 | 4.72 | 1.26 | -0.12 | NADP-dependent malic enzyme |
| Solyc06g007180.3 | AS | 5.73 | 5.52 | 1.44 | 1.23 | asparagine synthetase |
| Solyc08g080640.2 | AP24 | 6.77 | 6.08 | 1.11 | 0.41 | protein NP24 precursor |

**Table S3.** Expression of DEGs in different comparisons.

| **GO Term** | **Description** | **Color Model** | | | | **No: of DEGs** | |  |  |
| --- | --- | --- | --- | --- | --- | --- | --- | --- | --- |
|  |  | **R-3dpi vs. R-mock** | **S-3dpi vs. S-mock** | **R-mock vs. S-mock** | **R-3dpi vs. S-3dpi** | **R-3dpi vs. R-mock** | **S-3dpi vs. S-mock** | **R-mock vs. S-mock** | **R-3dpi vs. S-3dpi** |
| GO:0044699 | single-organism process | 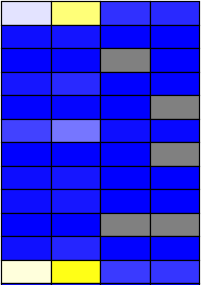  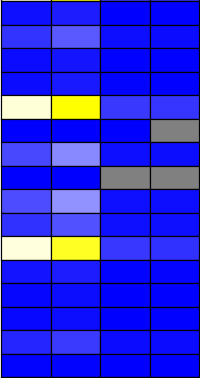  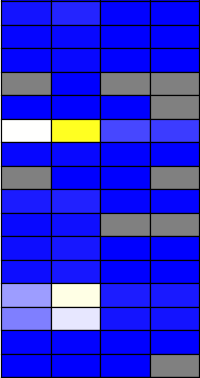 | | | | 559 | 955 | 120 | 102 |
| GO:0051704 | multi-organism process |  |  |  |  | 37 | 52 | 6 | 3 |
| GO:0040007 | growth |  |  |  |  | 7 | 15 |  | 2 |
| GO:0032502 | developmental process |  |  |  |  | 56 | 103 | 4 | 6 |
| GO:0002376 | immune system process |  |  |  |  | 8 | 15 | 2 |  |
| GO:0050896 | response to stimulus |  |  |  |  | 165 | 291 | 37 | 22 |
| GO:0040011 | locomotion |  |  |  |  | 2 | 4 | 1 |  |
| GO:0000003 | reproduction |  |  |  |  | 33 | 56 | 2 | 3 |
| GO:0022414 | reproductive process |  |  |  |  | 33 | 55 | 2 | 3 |
| GO:0022610 | biological adhesion |  |  |  |  | 1 | 2 |  |  |
| GO:0023052 | signaling |  |  |  |  | 41 | 92 | 8 | 6 |
| GO:0008152 | metabolic process |  |  |  |  | 711 | 1196 | 145 | 131 |
| GO:0032501 | multicellular organismal process |  |  |  |  | 39 | 72 | 3 | 5 |
| GO:0071840 | cellular component organization or biogenesis |  |  |  |  | 128 | 221 | 33 | 30 |
| GO:0048519 | negative regulation of biological process |  |  |  |  | 28 | 50 | 5 | 5 |
| GO:0048518 | positive regulation of biological process |  |  |  |  | 30 | 64 | 8 | 4 |
| GO:0009987 | cellular process |  |  |  |  | 722 | 1250 | 140 | 132 |
| GO:0098754 | detoxification |  |  |  |  | 2 | 3 | 1 |  |
| GO:0050789 | regulation of biological process |  |  |  |  | 178 | 336 | 33 | 31 |
| GO:0048511 | rhythmic process |  |  |  |  | 1 | 1 |  |  |
| GO:0065007 | biological regulation |  |  |  |  | 191 | 357 | 40 | 37 |
| GO:0051179 | localization |  |  |  |  | 119 | 200 | 40 | 38 |
| GO:0003824 | catalytic activity |  |  |  |  | 704 | 1164 | 138 | 121 |
| GO:0098772 | molecular function regulator |  |  |  |  | 48 | 71 | 8 | 12 |
| GO:0060089 | molecular transducer activity |  |  |  |  | 17 | 31 | 3 | 5 |
| GO:0004871 | signal transducer activity |  |  |  |  | 20 | 32 | 2 | 4 |
| GO:0005215 | transporter activity |  |  |  |  | 94 | 145 | 30 | 28 |
| GO:0045735 | nutrient reservoir activity |  |  |  |  | 6 | 7 | 1 | 1 |
| GO:0001071 | nucleic acid binding transcription factor activity |  |  |  |  | 48 | 90 | 6 | 5 |
| GO:0016209 | antioxidant activity |  |  |  |  | 15 | 21 | 2 | 2 |
| GO:0009055 | electron carrier activity |  |  |  |  | 13 | 21 | 4 | 1 |
| GO:0031386 | protein tag |  |  |  |  |  | 1 |  |  |
| GO:0000988 | transcription factor activity, protein binding |  |  |  |  | 2 | 3 | 1 |  |
| GO:0005488 | binding |  |  |  |  | 627 | 1167 | 177 | 149 |
| GO:0005198 | structural molecule activity |  |  |  |  | 17 | 21 | 5 | 5 |
| GO:0016530 | metallochaperone activity |  |  |  |  |  | 1 | 1 |  |
| GO:0005576 | extracellular region |  |  |  |  | 69 | 84 | 11 | 11 |
| GO:0099512 | supramolecular fiber |  |  |  |  | 22 | 38 |  |  |
| GO:0030054 | cell junction |  |  |  |  | 33 | 58 | 2 | 3 |
| GO:0055044 | symplast |  |  |  |  | 33 | 58 | 2 | 3 |
| GO:0016020 | membrane |  |  |  |  | 387 | 688 | 70 | 64 |
| GO:0044425 | membrane part |  |  |  |  | 313 | 567 | 51 | 48 |
| GO:0044421 | extracellular region part |  |  |  |  | 10 | 9 | 4 | 2 |
| GO:0031012 | extracellular matrix |  |  |  |  | 1 | 1 | 1 |  |
| GO:0044464 | cell part  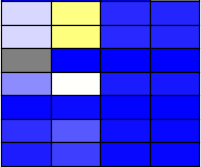 |  |  |  |  | 528 | 934 | 99 | 91 |
| GO:0005623 | cell |  |  |  |  | 529 | 940 | 99 | 91 |
| GO:0009295 | nucleoid |  |  |  |  |  | 1 | 2 | 2 |
| GO:0043226 | organelle |  |  |  |  | 344 | 632 | 69 | 57 |
| GO:0031974 | membrane-enclosed lumen |  |  |  |  | 14 | 28 | 6 | 5 |
| GO:0044422 | organelle part |  |  |  |  | 114 | 217 | 33 | 20 |
| GO:0032991 | macromolecular complex |  |  |  |  | 70 | 155 | 23 | 17 |

**Table S4.** Gene Ontology (GO) term enrichment analysis of DEGs in four comparisons.

Significance levels are based on enrichment and lowest FDR values with a cutoff of <0.05 that is indicated by color model mapping significance on a yellow to blue gradient scale. Highly significant terms indicated by yellow. — means no term assigned in that particular category.
